# Immune microenvironment in rotator cuff healing: from macrophage dynamics to immunomodulatory regenerative therapies

**DOI:** 10.3389/fbioe.2026.1990381

**Published:** 2026-10-16

**Authors:** Feng Li, Zhengdao Fu, Xinhai Zhang, Xiurong Ke, Xiang Gao

**Affiliations:** Department of Orthopaedic Surgery, Hangzhou Geriatric Hospital, Affiliated Hangzhou First People’s Hospital Chengbei Campus, School of Medicine, Westlake University, Hangzhou, China

**Keywords:** extracellular vesicles, immune microenvironment, immunomodulatory biomaterials, macrophage, mechanobiology, rotator cuff, tendon-to-bone healing

## Abstract

Rotator cuff repair is increasingly limited by biology rather than fixation alone: the repaired tendon commonly heals through fibrovascular scar rather than recreating the graded tendon-fibrocartilage-bone enthesis, particularly in aging, metabolic disease, muscle degeneration, and poor bone quality. The immune microenvironment is a dynamic determinant of whether injury progresses toward persistent inflammation and fibrosis or toward resolution and regeneration. This Review synthesizes immune regulation across the rotator cuff muscle-tendon-enthesis-bone unit, with emphasis on macrophage heterogeneity, efferocytosis, autophagy, immunometabolism, mechanosensing, and immune-stromal crosstalk. Human single-cell studies identify pro-fibrotic macrophage, senescent tenocyte, osteopontin/TGF-beta, and endothelial programs in rotator cuff scar, while mechanistic studies nominate resolution pathways as therapeutic targets. We critically evaluate cytokine/chemokine modulation, extracellular vesicles, senomorphic strategies, bioactive ions, ROS-responsive materials, nanoengineered interfaces, and rehabilitation-based immunomodulation. Across these approaches, the key translational requirements are causal mechanism, validation in chronic or aged disease models and human tissue, integration of structural and mechanical outcomes, arthroscopic deployability, manufacturing feasibility, and biomarker-guided patient selection. Together, these data support a shift from nonspecific anti-inflammatory augmentation toward mechanism-based, pro-resolution strategies for durable tendon-to-bone repair.

## Graphical Abstract

**Figure:**
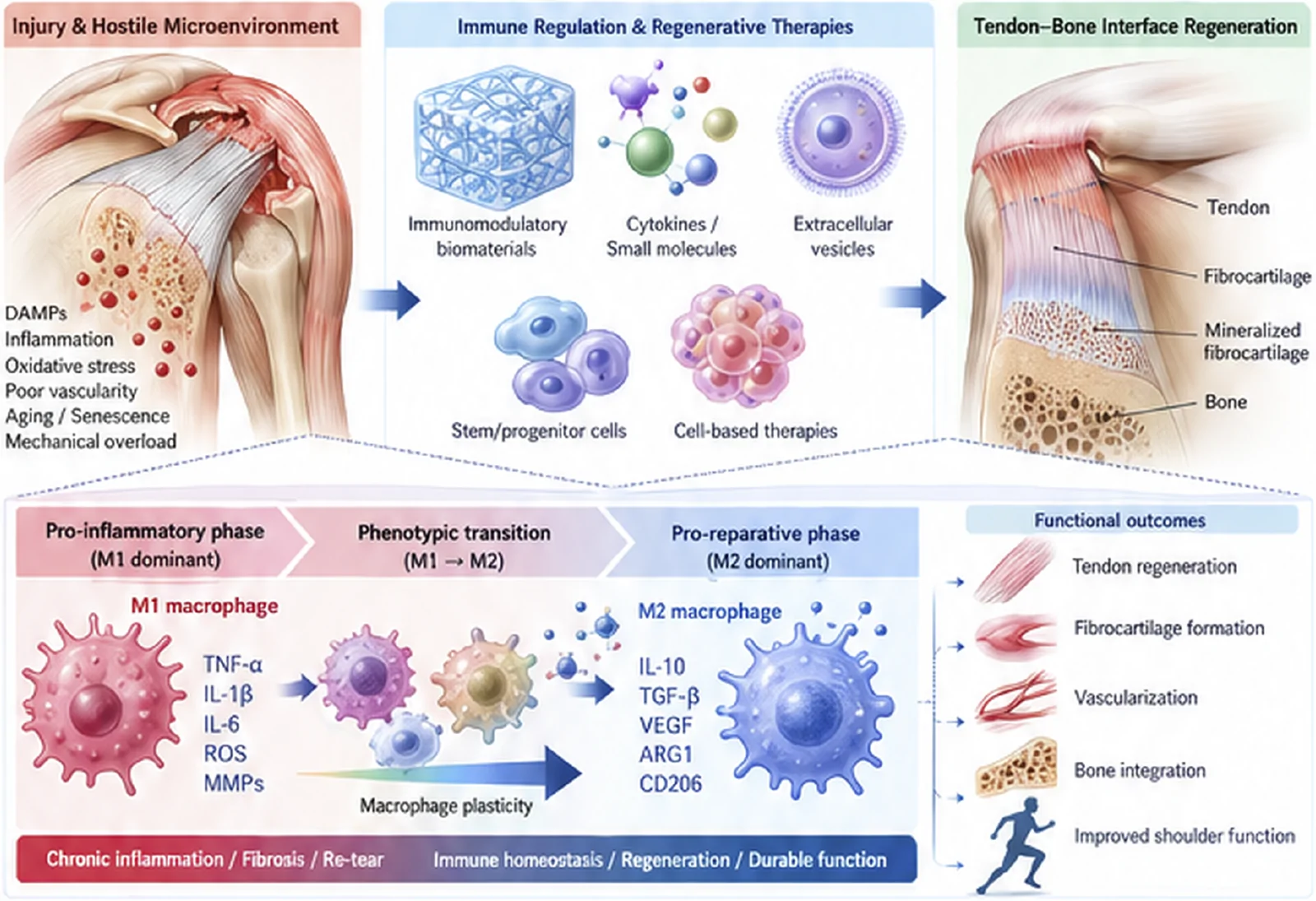
Conceptual framework linking the hostile post-tear microenvironment to immune-state transitions and immunomodulatory regenerative strategies, with the objective of restoring fibrocartilage, vascular support, bone integration, and durable shoulder function.

## 1 Introduction

Rotator cuff tears are among the most prevalent musculoskeletal disorders in older adults and remain a major cause of pain, weakness, and loss of shoulder function. Arthroscopic repair has become technically sophisticated, yet structural failure remains common in large, chronic, revision, and degenerative tears (Li D. et al., 2025; Liu et al., 2025; Qin et al., 2026; Li Y. et al., 2025). The central limitation is biological: the repaired supraspinatus tendon rarely regenerates the native graded enthesis and instead inserts through mechanically inferior fibrovascular scar. This mismatch persists even when time-zero fixation strength is excellent, indicating that durable success depends on the local healing environment rather than fixation alone (Derwin et al., 2010; Ricchetti et al., 2012; Chainani and Little, 2016; Longo et al., 2013). The tendon–bone interface denotes the physical junction between tendon and bone; the enthesis denotes its specialized, graded insertion with fibrocartilaginous zones. Tendon-to-bone healing denotes the biological repair process after reattachment, which may produce fibrovascular scar without recreating a native enthesis. We reserve tendon–bone for the interface or for a broad anatomical context, and use tendon-to-bone healing when discussing repair outcomes.

Historically, rotator cuff biology was framed primarily in terms of tendon cellularity, growth factors, stem/progenitor cells, extracellular matrix, and mechanical loading. These remain essential, but they are now understood to operate within an immune context that is both instructive and dynamic. Immune cells clear necrotic tissue, sense damage-associated molecular patterns (DAMP), shape angiogenesis, control matrix turnover, and alter lineage decisions in resident and recruited progenitors(Wang et al., 2023; Arvind et al., 2025). At the same time, excessive or unresolved inflammation promotes tenocyte senescence, matrix metalloproteinase activity, fibroblast activation, scar formation, fatty degeneration, and impaired bone-side integration (Crosio and Huang, 2022; Klatte-Schulz et al., 2024).

Macrophage responses vary with cell origin, tissue niche, metabolism, and injury phase; thus, M1/M2 markers are useful descriptors but insufficient endpoints for defining regenerative repair (Muscat et al., 2022; Smolyak et al., 2025; Ma et al., 2024; Meizlish et al., 2024; Ngai et al., 2023; Yu et al., 2022; Ma et al., 2023; Loffredo et al., 2024; Zhou et al., 2026; Yang et al., 2025). Accordingly, we assess immunomodulatory interventions by their effects on immune-state transitions, tissue-specific repair, and the timing and location of delivery, rather than by a single polarization marker (Liu et al., 2022; Wang et al., 2025; He et al., 2025; Li et al., 2024; He et al., 2026; Zheng et al., 2026; Cai et al., 2026; Liu Y. et al., 2026).

Recent reviews have already addressed macrophage polarization in tendon healing, immune regulation of tendon-to-bone repair, macrophage-directed biomaterials, MSC–immune interactions in rotator cuff repair, and rotator cuff exosome therapies (Li D. et al., 2025; Jiang et al., 2024; Gao et al., 2022; Wang Y. et al., 2024; Wang et al., 2026; Aratikatla et al., 2025). Table 1 compares their principal emphases with those of this Review. Our contribution is an integrative framework spanning the tendon stump, enthesis, muscle, bursa, and bone: it links human rotator cuff observations to mechanistic studies of heterogeneous immune states, efferocytosis and other resolution pathways, immunometabolism, stromal and vascular interactions, mechanics, extracellular vesicles, and biomaterials. We distinguish direct human evidence from animal and extrapolated evidence when identifying therapeutic design principles and testable translational gaps. These topics and concepts are not presented as individually new; the added value lies in their organization across anatomical niches, phases of repair, and levels of evidence.

**TABLE 1:** Comparison with closely related reviews. Entries describe the emphasis of each review, not whether a topic is mentioned at all.

Review and scope	RC specificity and human evidence	Macrophage states, resolution and metabolism	FAPs, bursa and mechanobiology	EVs, biomaterials and translation
Gao et al. (2022) tendon–bone interface	Broad tendon–bone; RC is among the applications; human RC evidence is not the organizing focus	Macrophage phenotypes and polarization are central; efferocytosis and immunometabolism are not organizing themes	Muscle FAPs and bursa are outside the main scope; mechanical interface is contextual	Immunomodulatory biomaterials are central; therapeutic challenges are discussed
Wang et al. (2024a) tendon healing	Primarily tendon injury and animal models; no focused human RC evidence synthesis	M1/M2 dynamics and adhesions are central; resolution pathways and metabolic state are not organizing themes	FAP and bursal niches are outside scope; loading is not integrated across RC niches	Includes selected EV and material strategies; clinical translation is discussed briefly
Jiang et al. (2024) tendon–bone healing	Broad tendon–bone focus, including RC examples; human RC studies are not stratified as a separate tier	Inflammatory cells and pathways are surveyed; efferocytosis and immunometabolism are not integrated as a central framework	Considers nonimmune cells and systemic factors; no joint RC muscle–bursa niche synthesis	Surveys EVs and biomaterials and discusses treatment and translational barriers
Li et al. (2025a) RC enthesis	RC-specific, with translational discussion; principal emphasis is MSC-based evidence	MSC–immune crosstalk and macrophage polarization are central; state heterogeneity and resolution are less developed	Muscle FAP and bursal niches are not the organizing scope; immune mechanics are secondary	MSC EVs and material-assisted delivery are substantial topics; patient variability is discussed
Aratikatla et al. (2025) RC exosomes	RC-specific systematic review of exosome studies; human/clinical work considered	Immune effects appear within an exosome treatment synthesis, rather than a full immune-state framework	FAPs, bursa and mechanical immune cues are not the organizing scope	Exosome evidence is the principal subject; other immunomodulatory modalities are outside scope
Wang et al. (2026) tendon–bone healing	Broad tendon–bone scope with RC examples; no separate human RC evidence hierarchy	Polarization and regulatory pathways are central; importantly, efferocytosis is also discussed	Multiple regulating factors are considered; RC FAP–bursa–muscle integration is not the main focus	Biomaterials and targeted macrophage interventions are major topics
Present Review whole RC repair unit	RC tendon, enthesis, muscle, bursa and bone; explicitly separates human RC findings from model and extrapolated evidence	Integrates heterogeneity, efferocytosis/resolution, immunometabolism and redox biology by niche and phase	Connects FAP and bursal biology with mechanical cues and immune–stromal communication	Integrates EVs, biomaterials, rehabilitation and evidence-calibrated translational criteria

“Human” denotes direct human rotator cuff tissue or clinical evidence; evidence may still be observational. RC, rotator cuff; FAP, fibro-adipogenic progenitor; EV, extracellular vesicle.

## 2 Scope and literature search strategy

This critical narrative review used PubMed as the documented electronic database; the last literature update was completed on 6 September 2026. Search concepts were combined in varying forms, including rotator cuff, tendon–bone interface, enthesis, macrophage, monocyte, neutrophil, regulatory T cell, dendritic cell, immune microenvironment, inflammation resolution, efferocytosis, autophagy, immunometabolism, senescence, fibro-adipogenic progenitor, subacromial bursa, extracellular vesicle, exosome, hydrogel, biomaterial, ROS-responsive, and mechanobiology. Reference lists of directly relevant primary studies and recent tendon-healing, immunology, extracellular-vesicle, and biomaterials reviews were additionally checked. Studies were selected for relevance to human rotator cuff pathology or repair, causal rotator cuff experiments, mechanistic work in other tendon/tendon–bone systems, or broader biology needed to explain a proposed mechanism. Direct human shoulder data and studies with a defined perturbation or structural/biomechanical endpoint were emphasized; landmark older studies were retained where necessary. This was purposive selection, with no prespecified eligibility checklist, duplicate screening, or formal risk-of-bias assessment. A reproducible search of Web of Science, Scopus, or Embase is not documented; consequently, PubMed-centered retrieval may have missed biomaterials and engineering studies indexed elsewhere.

To calibrate claims, we report three independent dimensions: (1) disease context—human rotator cuff tissue/clinical material, rotator cuff animal model, non-rotator-cuff tendon/tendon–bone model, or broader biology; (2) causal support—descriptive association, intervention with pathway readouts, or direct mechanistic perturbation; and (3) translational maturity—human descriptive/*ex vivo* observation, preclinical intervention, or clinical outcome testing. A human association does not establish causation, and an animal mechanism is not clinical validation. Tables 2–5 and the figure legends distinguish these dimensions.

**TABLE 2:** Functional immune-cell landscape during rotator cuff healing. Timing is approximate and cell states overlap.

Immune compartment	Typical window	Pro-repair functions	Failure mode if dysregulated	Evidence context
Neutrophils	Hours–days	Debris clearance; ROS/protease release; recruitment cues	Persistent tissue damage if unresolved	Broad tendon/RC model; limited human RC mapping Sources: (Crosio and Huang, 2022; Muscat et al., 2022; Smolyak et al., 2025; Chen et al., 2025c)
Monocytes/recruited macrophages	Days–weeks	Phagocytosis; cytokines; matrix regulation; stromal cross-talk	Persistent inflammatory or fibrotic signaling	RC models plus non-cuff tendon studies Sources: (Crosio and Huang, 2022; Muscat et al., 2022; Smolyak et al., 2025; Chen et al., 2025c)
Resident macrophages	Homeostasis–repair	Surveillance; matrix sensing; rapid local response	Loss of niche-specific repair programs	Homeostatic tendon and models; human RC state associations Sources: (Muscat et al., 2022; von Stade et al., 2025)
Dendritic cells	Early–intermediate	Antigen presentation; immune orchestration	Potential chronic immune activation	Non-cuff tendon EV study; endogenous RC role untested Sources: (Crosio and Huang, 2022; Chen et al., 2024a)
Regulatory T cells	Intermediate–resolution	Inflammation resolution; stromal repair signals	Insufficient resolution and tenocyte recruitment	Non-cuff tendon perturbation; adult RC causality untested Sources: (Arvind et al., 2025; Loffredo et al., 2024; Nayer et al., 2024; Dwyer et al., 2022; Ahn et al., 2025)
B cells/other lymphocytes	Chronic/incompletely defined	Cytokines, antibodies, antigen presentation	Unknown in rotator cuff; research gap	Human RC functional evidence lacking Sources: (Liu et al., 2025; Crosio and Huang, 2022)
FAP-associated immune niche	Chronic muscle degeneration	Macrophage–FAP EV signaling; adipogenic/fibrotic control	Fat expansion, fibrosis, persistent weakness	Human RC muscle and RC models; macrophage link partly inferred Sources: (Zhang et al., 2024c; Zhou et al., 2024; Agha et al., 2021; Lin et al., 2025; Zhang et al., 2025)

**TABLE 3:** Molecular circuits with particular relevance to immune regulation of rotator cuff and tendon-to-bone healing.

Circuit	Principal immune output	Therapeutic principle	Evidence context and causal support
TLR–NF-κB/MAPK	DAMP sensing; TNF-α, IL-1β, IL-6, MMPs	Early defense; limit persistence	Human RC cytokine associations; pathway-targeted RC model interventions (Wang et al., 2025; He et al., 2025) Sources: (Crosio and Huang, 2022; Klatte-Schulz et al., 2024; Yang et al., 2025; Wang et al., 2025; He et al., 2025; He et al., 2026; Zheng et al., 2026; Chen et al., 2025c)
NLRP3 inflammasome	IL-1β maturation; pyroptosis	Limit excessive sterile inflammation	Broader tendon/immunology plausibility; no RC-specific causal perturbation established by cited refs Sources: (Klatte-Schulz et al., 2024; Yang et al., 2025; Zhang et al., 2026; Jiang et al., 2024)
IL-4/IL-13–JAK/STAT6	Resolution-associated macrophage programs	Timed transition toward repair	RC animal intervention with pathway readouts (Liu et al., 2022; Chen et al., 2025a); not a human causal test Sources: (Liu et al., 2022; Chen et al., 2025a)
TGF-β/SMAD	Chondrogenesis and matrix deposition; fibrosis	Spatial/temporal control	Human RC scar association (Qin et al., 2026) plus murine RC macrophage-source perturbation (Wang et al., 2023) Sources: (Qin et al., 2026; Wang et al., 2023; Li et al., 2023)
CCL2–CCR2	Monocyte/macrophage recruitment	Avoid both excessive influx and complete depletion	Tendon resident/recruited-cell and pharmacological studies; RC-specific inference limited (Muscat et al., 2022; Smolyak et al., 2025) Sources: (Muscat et al., 2022; Smolyak et al., 2025)
ROS–HIF-1α/Nrf2	Redox stress, immunometabolic state	ROS-scavenging and responsive delivery	Preclinical tendon–bone/RC material response; RC immune-metabolic causality varies by study Sources: (Yu et al., 2022; He et al., 2026; Zheng et al., 2026; Cai et al., 2026; Liu et al., 2026b; Voss et al., 2021; Nakamura et al., 2026; Huo et al., 2026)
cGAS–STING	Mitochondrial/DNA stress sensing	Mitochondrial rescue and innate immune control	Achilles animal intervention (Liu et al., 2026b); extrapolated to RC. Sources: (Liu et al., 2026b)
IL-33/Treg	Type-2 transition and repair regulation	Coordinate adaptive and innate immunity	Neonatal non-cuff tendon perturbation (Arvind et al., 2025); extrapolated to adult RC. Sources: (Arvind et al., 2025; Loffredo et al., 2024; Dwyer et al., 2022; Ahn et al., 2025)
Efferocytosis	Inflammation resolution and metabolic rewiring	Promote apoptotic-cell clearance	Human RC bursal mediators (Klatte-Schulz et al., 2024) indicate resolution potential, not efferocytosis; ACL tendon–bone perturbation (Yao et al., 2026) and other non-cuff tendon–bone work (Zhao et al., 2026) support mechanism Sources: (Ngai et al., 2023; Ma et al., 2023; Li et al., 2026; Yao et al., 2026; Zhao et al., 2026)

**TABLE 4:** Representative immune-regenerative strategies with separate disease context, causal support, and translational maturity.

Strategy	Immune mechanism	Representative outcome	Disease context	Causal support	Translational maturity
Mechanical stimulation (Liu et al., 2022)	IL-4/JAK/STAT; macrophage state transition	Improved tendon–bone healing in mouse rotator cuff repair (RCR)	RC animal model	Animal intervention; pathway tested	Preclinical only
Leptin pathway (Li et al., 2025c)	TNF-associated inflammatory macrophage polarization	Impaired rotator cuff repair healing; identifies metabolic-risk target	RC animal model	Animal intervention; pathway tested	Preclinical only
Inflammation-responsive micro-hydrogel (Chen et al., 2025a)	SDF-1 recruitment + MMP-responsive IL-4	MSC homing and macrophage immunomodulation	RC animal model	Animal intervention; pathway tested	Preclinical only
Lactic-acid dynamic hydrogel (Wang et al., 2025)	NF-κB inhibition + VEGF	Anti-inflammatory and pro-vascular rotator cuff repair	RC animal model	Animal intervention; pathway readouts	Preclinical only
Mg nanoflower hydrogel (Li et al., 2024)	Authors’ M2-associated marker shift; Mg2+/procyanidin release	Fibrocartilage and mineral regeneration	Other tendon/tendon–bone model	Animal intervention; pathway readouts	Preclinical only
3D-printed Mg/PCL scaffold (Wang et al., 2024a)	CD11b+CD206+ increase and CD11b+CD86^+^ decrease; osteogenesis (Wang et al., 2024a)	Improved RCR tissue ingrowth and inflammation control	RC animal model	Animal intervention; pathway readouts	Preclinical only
Mn-silicate multicellular scaffold (Du et al., 2024)	PGE2-dependent macrophage regulation	Integrated tendon–bone regeneration	Other tendon/tendon–bone model	Animal intervention; pathway readouts	Preclinical only
ROS-triggered adhesive (He et al., 2026)	Redox-responsive cargo release	Inflammation control + lineage differentiation	Other tendon/tendon–bone model	Animal intervention; pathway readouts	Preclinical only
ROS-responsive nanocomposite hydrogel (Zheng et al., 2026)	NF-κB suppression + redox control	Collagen remodeling, osteogenesis, chondrogenesis	Other tendon/tendon–bone model	Animal intervention; pathway readouts	Preclinical only
Ce-bioactive-glass suture interface (Cai et al., 2026)	ROS scavenging; HIF-1α-linked immunometabolic shift	Fibrocartilage and bone integration	RC rat repair model	Animal intervention; pathway readouts	Preclinical only
EPO nanoparticles (Liu et al., 2026b)	Mitochondrial rescue; cGAS–STING inhibition	Macrophage reprogramming and tendon regeneration	Other tendon/tendon–bone model	Preclinical intervention; mechanism varies	Preclinical only
Healthy TSPC exosomes (Zhang et al., 2024c)	Break senescent TSPC–macrophage feedback	Aged chronic RCR healing	RC animal model	Animal intervention; pathway tested	Preclinical only
TSPC exosomes (He et al., 2024)	miR-21a-5p/PDCD4; macrophage regulation	Improved RCR histology and biomechanics	RC animal model	Animal intervention; pathway tested	Preclinical only
SHED exosome-mimetics (He et al., 2025)	Autophagy activation; NF-κB suppression	Multilineage tendon–bone interface (TBI) regeneration	RC animal model	Animal pathway perturbation	Preclinical only
PRP-derived exosomes (Han et al., 2024)	Cell-free paracrine modulation	Improved tendon–bone healing	RC animal model	Animal intervention; pathway readouts	Preclinical only
HyA hydrogel in delayed RCR (Pfaff et al., 2025)	FAP regulation; IL-10; vascularization	Reduced muscle fibrosis/fat and improved quality	RC animal model	Animal intervention; pathway readouts	Preclinical only
specialized pro-resolving mediators (SPM)/bursal resolution pathways (Klatte-Schulz et al., 2024)	Active inflammation resolution	Human rotator cuff disease mechanistic target	Human RC tissue	Descriptive/*ex vivo* association	Human observational/*ex vivo*; not efficacy
Mg2+/curcumin self-healing hydrogel (Chen et al., 2021)	Anti-inflammatory + pro-differentiation	Rabbit rotator cuff TBI regeneration	RC animal model	Animal intervention; pathway readouts	Preclinical only
Osteoporotic EV/hydrogel systems (Song et al., 2023; Kong et al., 2026)	Disease-specific immune and stromal reprogramming	Osteoporotic tendon–bone healing	Tendon–bone and osteoporotic RC rat models	Preclinical intervention; mechanism varies	Preclinical only
Primary human macrophage-tenocyte EV system (von Stade et al., 2025)	Cell-of-origin-dependent extracellular-vesicle signaling	Human primary-cell evidence that EV products are mechanistically non-interchangeable	Human tendon primary cells; RC origin unclear	*Ex vivo* intervention; no patient outcome	Human *ex vivo*; no clinical efficacy

Human tissue findings and primary-cell assays are not clinical efficacy data. “Pathway tested” indicates mechanistic measurements or selected pathway interventions in the cited model; only a specific blocking or loss-of-function experiment warrants a stronger causal claim.

**TABLE 5:** Translational readiness of representative rotator cuff immunomodulatory approaches.

Strategy and refs	Model; chronic or aged test	Structural and mechanical readouts	Human and clinical evidence	Safety; arthroscopy; manufacture
PRP (Rossi et al., 2024; Yao et al., 2024)	Human repair RCTs; effects in large chronic/aged tears not established by these trials	MRI retears and patient function assessed; trials disagree on structural benefit; no direct cuff strength test	Clinical RCT evidence; immune mechanism in repaired human tissue unproven	Arthroscopic injection demonstrated; report leukocytes, activation, dose and lot variation; pain/stiffness and infection monitored
Acellular collagen patches (Hurley et al., 2024; Warren et al., 2024; de Andrade et al., 2022)	Human rotator cuff surgical studies; specific chronic/aged immune-response testing incomplete	Imaging retear and clinical function evaluated; no direct *in vivo* enthesis mechanics in patients	Clinical outcome literature exists; immune mechanism not established	Arthroscopic patch placement established for selected products; integration, inflammatory reaction, source, processing and sterility are product-specific
MSC or bursal-cell adjuncts (Utsunomiya et al., 2013; Hernigou et al., 2014; Meyer et al., 2015; Eliasberg et al., 2017; Steinert et al., 2015)	Mixed preclinical cuff models and selected human shoulder use; chronic/aged immune efficacy not established	Model histology/mechanics vary by product; clinical structural evidence is limited and heterogeneous	Human tissue/cell studies and limited clinical literature; specific immune mediation unproven	Arthroscopic handling depends on preparation; cell identity, viability, donor age, sterility and potency need control
Tendon-stem-cell EVs (Zhang et al., 2024c; He et al., 2024)	Rat rotator cuff repair; (Zhang et al., 2024c) explicitly tests aged chronic tears, (He et al., 2024) rat repair	Interface histology and mechanics in models; no patient retear endpoint	Human tendon-cell assays inform plausibility (von et al., 2025); no RC therapeutic trial	Local delivery experimentally feasible; EV identity, cargo, potency, purity, storage, sterility and retention unresolved
Senomorphic EV hydrogel (Kong et al., 2026)	Osteoporotic rat rotator cuff repair; not equivalent to a chronic aged tear	Fibrocartilage, bone and ultimate load/stiffness assessed in model	No human cuff efficacy; mechanistic cell assays do not replace patient validation	Test stage-dependent senescence effects, off-target cargo and ectopic bone; injection shown in rat, portal handling and GMP scale-up unproven
ROS-responsive hydrogel (Huo et al., 2026)	Rat rotator cuff tear repair; chronic/aged and diabetic models not established in cited study	Fibrocartilage/collagen organization and biomechanical strength reported	No human cuff therapeutic validation or clinical trial	ROS threshold and excess scavenging need study; adhesion under irrigation, sterilization, release stability and reproducibility unproven
ROS-scavenging suture (Cai et al., 2026)	Rat rotator cuff model; chronic/aged testing not established in cited report	Fibrocartilage and bone microstructure reported; cyclic fatigue/clinical integrity unproven	No human cuff therapeutic validation or clinical trial	Suture route aligns with arthroscopy in principle; coating retention, particle release, fatigue, sterilization and lot consistency require testing
Delayed-repair HyA hydrogel (Pfaff et al., 2025)	Mouse delayed repair (6 weeks); tests chronicity proxy, not aged human disease	Muscle fibrosis, fat and vascularity evaluated; enthesis fatigue and patient function untested	Human FAP culture included; no human therapy outcome	Local application in mouse; portal delivery, retention, degradation products and sterile manufacturing need work
Immunometabolic EPO system (Liu et al., 2026b)	Achilles tendon preclinical model; human cuff and chronic/aged cuff untested	Tendon model regeneration and mitochondrial markers; cuff enthesis mechanics unknown	No rotator cuff clinical evidence	Off-target metabolic/hematologic effects and nanoparticle distribution require assessment; arthroscopic use and product scale-up untested

“Not established” means the cited work does not demonstrate the criterion; it does not prove that the test has never been performed. RCT, randomized controlled trial; EV, extracellular vesicle; HyA, hyaluronic acid; GMP, good manufacturing practice.

The recency target is intentional because the field has changed rapidly with the arrival of human single-cell atlases, immune-metabolic studies, senescence-targeted therapies, responsive biomaterials, and macrophage-directed extracellular-vesicle systems. Landmark older studies are retained where they define stem/progenitor-cell biology, tendon-bone mechanics, scaffolding concepts, or the historical path from augmentation to regenerative engineering.

## 3 The immune microenvironment as a multiscale determinant of rotator cuff healing

### 3.1 Sterile injury, DAMP sensing, and the initiation of repair

Tendon detachment and surgical repair create a sterile wound with necrotic matrix, microvascular disruption, hematoma, suture-related injury, and altered mechanical strain. DAMP released from damaged cells and extracellular matrix fragments engage pattern-recognition receptors on resident macrophages, infiltrating monocytes, dendritic cells, and stromal cells. Toll-like receptor signaling, NF-κB activation, inflammasome priming, and the release of IL-1β, TNF-α, IL-6, chemokines, prostaglandins, and reactive oxygen species initiate leukocyte recruitment and matrix breakdown (Crosio and Huang, 2022; Yang et al., 2025; Voss et al., 2021; Shen et al., 2021). This early response is not intrinsically pathological; it establishes antimicrobial competence, clears debris, and activates repair. The problem is persistence, inappropriate amplitude, or failure to transition toward resolution (Klatte-Schulz et al., 2024; Chen R. Q. et al., 2025).

The rotator cuff differs from a simple midsubstance tendon wound because the surgical target is a soft-to-hard interface. The repaired footprint contains tendon, fibrocartilaginous remnants, cortical and cancellous bone, marrow-derived cells, suture material, and often microfracture-related bleeding (Wang et al., 2023; Schulze-Tanzil et al., 2022). Each compartment presents different stiffness, oxygen tension, ion composition, and cell populations. Consequently, the same cytokine may be reparative in one location and deleterious in another. For example, early TNF and IL-1 signaling can recruit and activate phagocytes, whereas prolonged exposure suppresses matrix synthesis and favors catabolic remodeling. Similarly, TGF-β supports matrix deposition and chondrogenesis but can drive myofibroblast activation and fibrosis when excessive or spatially uncontrolled (Rodeo et al., 2007; Kovacevic and Rodeo, 2008; Seeherman et al., 2008; Kabuto et al., 2015; Tokunaga et al., 2015).

### 3.2 Spatial immune niches: tendon stump, enthesis, muscle, bursa, and bone

A key conceptual advance is that there is no single ‘rotator cuff immune microenvironment.’ At least five interacting niches can be distinguished. The tendon stump contains tenocytes, tendon stem/progenitor cells (TSPCs), stromal cells, resident immune cells, and degenerative extracellular matrix (Qin et al., 2026; Liu et al., 2023). The enthesis couples tendon, fibrocartilage, mineralized fibrocartilage, and bone, exposing immune cells to abrupt gradients in stiffness, mineral, oxygen tension, and cell lineage (Li et al., 2024; He et al., 2026). The muscle compartment contributes macrophages, fibro-adipogenic progenitors (FAPs), denervation-associated signals, fibrosis, and lipid-rich metabolic cues. The subacromial bursa is not inert tissue; it can act as an inflammatory and pro-resolving signaling organ. Finally, the humeral footprint and marrow provide osteoimmune interactions involving monocytes, macrophages, osteoclast-lineage cells, BMSCs, and vascular cells. These compartments communicate, but the same cytokine or macrophage program can be beneficial in one location and harmful in another (Ju et al., 2024).

Human data are beginning to move this framework beyond inference. Single-cell analysis of rotator cuff tendon stumps identified heterogeneous scar-associated stromal and immune populations, including senescent tenocytes, SOX9-associated pro-fibrotic macrophages, and endothelial cells undergoing endothelial-to-mesenchymal transition; osteopontin and TGF-beta signaling emerged as central fibrotic nodes (Qin et al., 2026). Separately, transcriptomic profiling of subacromial bursa showed that degenerative rotator cuff tears carry a distinct inflammatory signature with macrophage heterogeneity compared with traumatic tears and proximal humeral fracture controls. These observations show that the human disease niche contains cell states that are poorly reproduced by an acute tendon detachment in a young rodent.

The bursa deserves similar attention. Human bursal tissue contains cytokines and specialized pro-resolving mediators, and its transcriptomic program changes with degenerative disease (Klatte-Schulz et al., 2024). This makes the bursa a potential source of both pathological and regenerative signals rather than simply tissue to be removed during decompression. A high-impact research priority is therefore to map whether bursal macrophage, stromal, vascular, and lipid-mediator programs predict tendon quality, pain phenotype, or postoperative healing - and whether preserving or selectively modulating the bursa is biologically advantageous.

### 3.3 Human evidence anchors: what is established in the diseased shoulder?

The strongest direct human evidence for an immune contribution to rotator cuff disease comes from a small but growing set of tissue and primary-cell studies. The 2026 single-cell atlas of rotator cuff scar provides direct evidence that macrophages are not a homogeneous M1/M2 pool: a SOX9-associated fibrotic macrophage state coexists with senescent tenocytes and pro-fibrotic endothelial programs, and osteopontin/TGF-beta signaling links these populations to extracellular-matrix deposition (Qin et al., 2026). Human subacromial bursa transcriptomics similarly demonstrates that degenerative tears have an inflammatory program distinct from traumatic tears, with macrophages implicated as major contributors (Ju et al., 2024). Human bursal studies also identify pro-resolving lipid mediators, supporting the idea that the shoulder contains endogenous resolution pathways as well as inflammatory ones (Klatte-Schulz et al., 2024). Separately, primary human macrophage-tenocyte experiments show that extracellular-vesicle effects depend on donor-cell origin, underscoring the need to treat cell products as mechanistically distinct rather than interchangeable (von Stade et al., 2025). The donor site and disease relevance of primary human macrophage–tenocyte EV experiments (von Stade et al., 2025) should not be assumed to establish rotator cuff-specific clinical evidence.

Human rotator cuff observations are presently dominated by cross-sectional tissue, primary-cell, and transcriptomic studies; findings from the diseased shoulder do not automatically predict postsurgical healing or demonstrate that manipulating the implicated pathway will help patients. Longitudinal within-patient sampling, spatial mapping of tendon edge, footprint, bursa, and muscle, *ex vivo* perturbation, and outcome linkage are required to advance beyond association.

Known in human rotator cuff tissue: scar and bursa studies identify heterogeneous immune/stromal states and pro-resolving mediators (Qin et al., 2026; Klatte-Schulz et al., 2024; Ju et al., 2024). Inferred or unvalidated: causal roles in retear prevention, optimal efferocytosis or adaptive-immune interventions, and patient efficacy of macrophage-directed EVs or biomaterials. Human tendon primary-cell EV experiments (von Stade et al., 2025) provide *ex vivo* mechanistic context but are not evidence of efficacy or confirmed rotator cuff donor specificity.

### 3.4 Temporal phases: inflammation must resolve, not disappear

The phases of healing overlap: early neutrophil and monocyte activity supports clearance and recruitment, whereas persistent inflammatory signaling can impair matrix remodeling (Arvind et al., 2025; Muscat et al., 2022; Liu et al., 2022). The identity, location, and kinetics of cells mediating the transition remain incompletely defined in human rotator cuff repair.

For therapy design, the key unanswered question is which intervention window preserves early repair functions while promoting subsequent tissue-specific remodeling (Figure 1) (Wang et al., 2025; Li et al., 2024; He et al., 2026; Zheng et al., 2026; Cai et al., 2026; Cheng et al., 2025).

**FIGURE 1:**
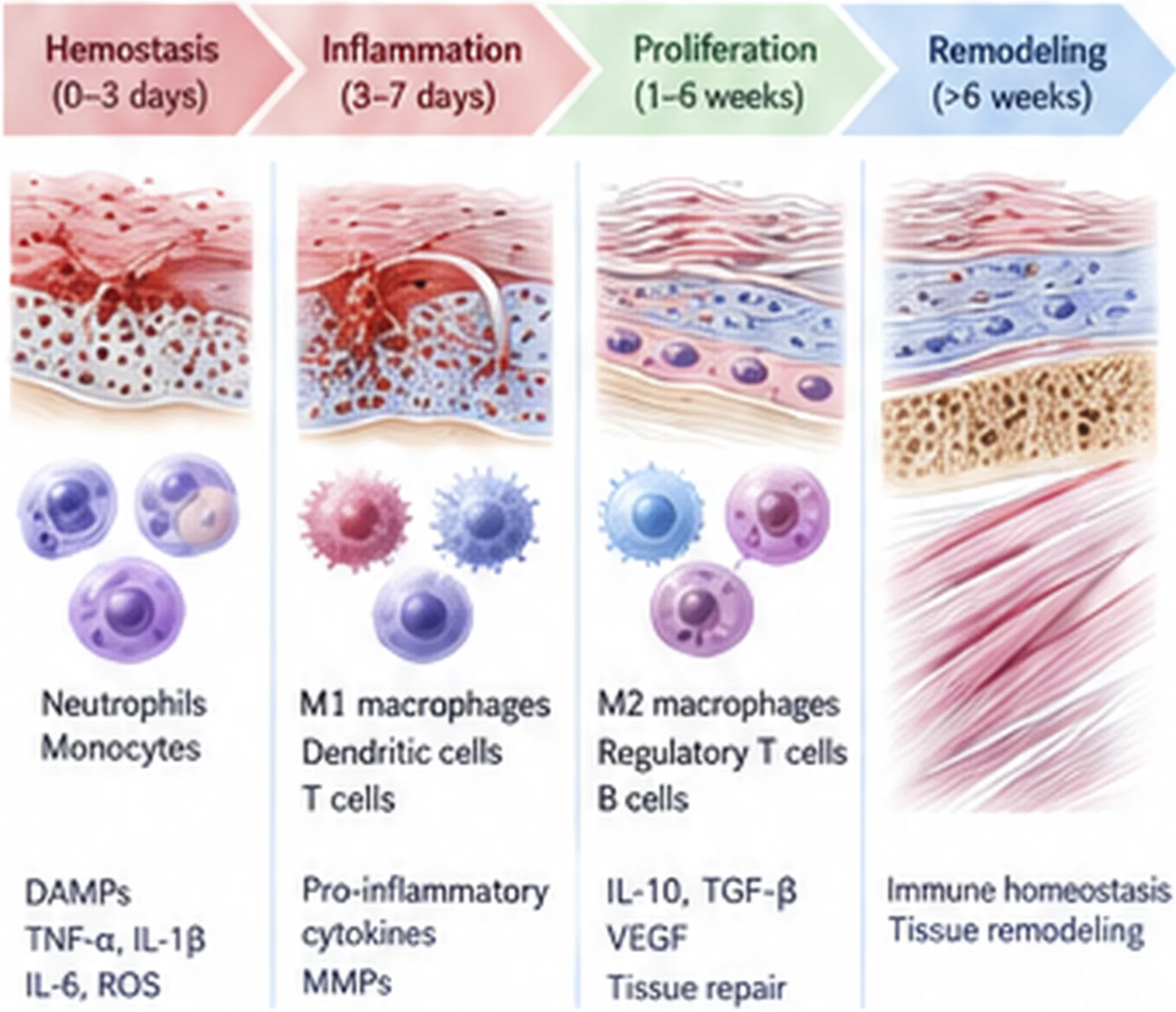
Spatiotemporal immune response after rotator cuff injury, illustrating a proposed transition from early inflammatory activity to pro-resolving immunity and tissue remodeling.

## 4 Immune-cell landscape of rotator cuff healing

### 4.1 Neutrophils and inflammatory monocytes: necessary initiators with a narrow therapeutic window

Neutrophils are among the earliest cells recruited to injured connective tissue. They release proteases, ROS, cytokines, and extracellular traps and can influence subsequent macrophage recruitment (Muscat et al., 2022). Their contribution to tendon repair is less well resolved than that of macrophages, but available evidence supports a dual role: early antimicrobial and debridement functions are beneficial, whereas persistent neutrophil activity can prolong matrix damage. Monocytes recruited through chemokine axes such as CCL2–CCR2 differentiate into macrophages and contribute to both inflammatory and reparative pools (Chen R. Q. et al., 2025).

The CCR2 story illustrates why simple depletion strategies can be misleading. Tendon-resident CCR2-positive macrophages and T cells are present even during homeostasis, and genetic CCR2 deficiency can impair healing because both resident and recruited macrophage participation is blunted. Conversely, pharmacological modulation of excessive CCR2-positive recruitment at selected time points may reduce fibrotic macrophage persistence (Smolyak et al., 2025). Thus, therapeutic value depends on dose, timing, and whether the target is recruited monocytes, resident macrophages, or both.

### 4.2 Macrophages: sensors, effectors, and organizers of the repair niche

Macrophages are uniquely positioned to integrate biochemical and biophysical information (Gao et al., 2022). They phagocytose necrotic tissue, secrete inflammatory and pro-resolving mediators, remodel matrix, regulate angiogenesis, communicate with fibroblasts and progenitor cells, and respond to oxygen tension, metabolites, extracellular-matrix stiffness, and implant surfaces. In tendon healing, macrophage depletion or uncontrolled inflammatory persistence frequently worsens functional outcomes, whereas appropriately timed pro-resolving states are associated with improved collagen organization and reduced scar (Ma et al., 2023; Yang et al., 2025).

Rotator cuff animal studies provide direct mechanistic evidence that macrophage state can influence structural repair. Mechanical stimulation can activate IL-4/JAK/STAT signaling and increase the authors’ M2-associated marker panel while improving tendon-bone integration (Liu et al., 2022). TGF-beta1 derived from macrophages contributes to load-dependent chondrogenesis at the repair interface (Wang et al., 2023). Leptin can bias macrophages toward a TNF-associated inflammatory marker program (termed M1 in the original study) through TNF signaling and impair tendon-to-bone healing, providing a plausible molecular link between obesity, fatty degeneration, systemic metabolism, and local repair. Together, these studies support a model in which macrophages translate systemic and local cues into structural outcomes.

### 4.3 Dendritic cells, mast cells, and other innate populations

Dendritic cells can link innate sensing to adaptive responses, but their function in rotator cuff repair has not been established. Dendritic-cell-derived exosomes have modulated macrophages in non-rotator-cuff tendon experiments (Chen R. et al., 2024); they do not demonstrate an endogenous dendritic-cell role in the human repaired shoulder. Mast-cell mediators and innate lymphoid-cell programs provide additional hypotheses based mainly on broader tendon or tissue biology (Crosio and Huang, 2022; Voss et al., 2021). These populations should be mapped rather than assigned a reparative role in rotator cuff disease by analogy.

### 4.4 T cells and regulatory T cells: emerging regulators of regeneration

Evidence for adaptive immunity is much thinner than for macrophages in rotator cuff repair. Tendon-resident T cells and pro-repair Treg–stromal interactions have been described mainly in non-rotator-cuff systems (Nayer et al., 2024; Dwyer et al., 2022; Ahn et al., 2025). Neonatal tendon perturbation experiments implicate Tregs and IL-33 in functional regeneration (Arvind et al., 2025); their necessity, timing, and target cells after adult human rotator cuff repair have not been demonstrated.

These studies motivate, but do not yet validate, therapies that recruit Tregs or coordinate T cells with macrophages in rotator cuff repair (Zhou et al., 2026; Shen et al., 2021; Nayer et al., 2024; Dwyer et al., 2022; Nie et al., 2024). This distinction should guide future studies toward rotator cuff-specific perturbation and postoperative outcomes.

### 4.5 B cells and humoral signals

B-cell states and functions after rotator cuff repair remain undefined. Cytokine release, antigen presentation, and antibody responses are biologically plausible functions, but their contribution to cuff repair cannot currently be inferred from their presence in other tissues (Liu et al., 2025; Crosio and Huang, 2022). Direct shoulder-tissue profiling and perturbation studies are needed before assigning benefit or harm.

### 4.6 FAP-associated immune niche

Fibro-adipogenic progenitors (FAPs) represent an emerging stromal component of the immune microenvironment in chronic rotator cuff degeneration. Following rotator cuff injury, FAP expansion and phenotypic remodeling are closely associated with fatty infiltration and fibrosis of the muscle, while bidirectional signaling between FAPs and macrophages may determine whether the injured niche progresses toward regeneration or persistent fibro-adipogenic remodeling (Zhang X. et al., 2024; Zhou et al., 2024). Recent studies further suggest that extracellular-vesicle-mediated communication participates in macrophage–FAP crosstalk, linking inflammatory signaling to adipogenic and fibrotic programs (Agha et al., 2021; Lin et al., 2025). Thus, the FAP-associated niche should not be viewed solely as a stromal consequence of chronic injury, but as an immune–stromal signaling compartment that may contribute to persistent muscle degeneration and weakness after rotator cuff tears (Zhang et al., 2025). Therapeutic strategies that modulate macrophage–FAP communication, rather than indiscriminately eliminating FAPs, may therefore provide a route to limit pathological fatty infiltration and fibrosis while preserving their potential reparative functions.

Human rotator cuff tissue supports macrophage heterogeneity and bursal immune signatures (Qin et al., 2026; Ju et al., 2024), but causal roles for B cells, dendritic cells, mast cells, and Tregs in adult human cuff healing remain untested. Rotator cuff model perturbations are more developed for macrophages (Li Y. et al., 2025; Wang et al., 2023; Liu et al., 2022; He et al., 2025) than for these other populations; neonatal or non-cuff tendon results (Arvind et al., 2025; Chen R. et al., 2024) are hypotheses for the repaired shoulder.

## 5 Macrophage dynamics beyond the M1/M2 paradigm

### 5.1 Why the binary model is useful—and why it is no longer enough

Markers conventionally grouped as M1 (such as CD86/iNOS) and M2 (such as CD206/ARG1) remain practical, study-specific descriptors in experimental rotator cuff studies, but they do not define two fixed *in vivo* endpoints. Macrophage programs vary with origin, cytokines, lipid and redox states, phagocytic cargo, mechanics, oxygen, and time (Crosio and Huang, 2022; Voss et al., 2021; Wang Y. et al., 2024; Li et al., 2026).

Where a cited experiment reports “M1” or “M2” polarization, we retain that designation only as the investigators’ marker-based terminology; it should not be read as a stable macrophage lineage or a demonstrated reparative function. We specify the measured pathway, marker, transcriptional state, or clearance function when available, and evaluate repair separately by tissue and mechanical outcomes.

A polarization-marker change alone cannot establish regeneration: prolonged TGF-β-associated activity may accompany fibrosis, and suppressing inflammatory functions can compromise clearance. Studies should therefore pair state profiling with functional assays and tissue-specific repair outcomes.

### 5.2 Ontogeny and macrophage heterogeneity

Macrophage populations in tendon arise from both tissue-resident cells and recruited monocytes. Resident cells may be adapted to local matrix architecture and provide rapid surveillance, whereas recruited monocytes expand the inflammatory and reparative response after injury. CCR2 studies indicate that the distinction is not absolute: CCR2-positive cells can exist in homeostatic tendon, and both resident and circulating pools contribute to repair (Muscat et al., 2022). Future lineage-tracing studies in rotator cuff models are needed to determine whether regenerative and fibrotic macrophage states preferentially arise from different origins.

Human single-cell data add another layer. Scar-associated macrophages after rotator cuff tear show transcriptional heterogeneity that cannot be captured by CD86-versus-CD206 staining alone. In particular, a SOX9-associated pro-fibrotic macrophage program co-localizes with osteopontin/TGF-beta activity and matrix deposition (Qin et al., 2026). This is a crucial conceptual shift: macrophages can acquire stromal-like or fibrosis-supporting programs without fitting neatly into canonical M1 or M2 categories. Future rotator cuff studies should therefore report cell origin, transcriptional state, spatial location, and function, and should use M1/M2 markers only as a low-resolution layer of description rather than the endpoint of mechanism.

### 5.3 Efferocytosis and inflammation resolution

Efferocytosis - the clearance of apoptotic cells by phagocytes - is a central mechanism of active inflammation resolution. General immunology establishes that successful efferocytosis limits secondary necrosis and DAMP release while inducing lipid, metabolic, and transcriptional programs that support pro-resolving macrophage expansion (Ngai et al., 2023; Ma et al., 2023). Evidence from a mouse anterior cruciate ligament reconstruction model supports this mechanism at a tendon–bone interface: low-intensity mechanical stimulation was reported to drive macrophage efferocytosis through ACSL4 lipid remodeling and CCL9-CCR1 signaling and to improve tendon-to-bone healing (Yao et al., 2026), while ZEB1-dependent control of mitochondrial fission and MFN2 was linked to macrophage efferocytosis and reduced inflammation in tendon-to-bone healing (Zhao et al., 2026). These are mechanistic tendon–bone studies, not direct evidence that human rotator cuff macrophages clear apoptotic cells in the same way (Yao et al., 2026; Zhao et al., 2026).

Efferocytosis therefore offers a more mechanistically precise therapeutic target than generic 'M2 polarization.’ Potential intervention points include apoptotic-cell recognition, mitochondrial dynamics, lipid remodeling, phagolysosomal processing, and the chemokine signals released after successful clearance. The translational challenge is timing: augmenting efferocytosis too early or too broadly could alter host defense, whereas delayed enhancement may fail to prevent secondary necrosis. Rotator cuff-specific studies should define when apoptotic burden peaks, which macrophage subsets perform clearance, and whether efferocytosis can be increased without suppressing the early inflammatory functions required for repair (Ngai et al., 2023; Ma et al., 2023).

### 5.4 Autophagy and intracellular quality control

Autophagy regulates macrophage survival, inflammasome activity, mitochondrial quality, and inflammatory signaling. It also intersects with tendon-cell and FAP biology. Stem cells from human exfoliated deciduous teeth (SHED)-derived exosome-mimetics can suppress NF-κB signaling and activate macrophage autophagy in a rotator cuff model, while inhibition of autophagy diminishes the anti-inflammatory effect (He et al., 2025). In parallel, metformin reduces FAP adipogenesis after rotator cuff tear through mTOR/ULK1-associated autophagy (Zhou et al., 2024). These observations suggest that autophagy is not merely a macrophage-intrinsic pathway but a cross-compartment regulator linking immunity to muscle quality and progenitor fate (Wang et al., 2026).

### 5.5 Immunometabolism and redox control

General immunometabolism literature establishes that macrophage phenotype is inseparable from metabolic state. Inflammatory programs commonly rely more heavily on glycolytic flux and generate inflammatory metabolites, whereas reparative states are often associated with oxidative metabolism and fatty-acid utilization, although these distinctions are context dependent (Ngai et al., 2023). Lactate generated during efferocytosis can support expansion of pro-resolving macrophages, while mitochondrial dysfunction can sustain inflammatory signaling. These principles provide biological plausibility for rotator cuff immunometabolic interventions, but should not be interpreted as direct human rotator cuff evidence.

Tendon and rotator cuff biomaterial studies are beginning to exploit immunometabolic and redox control. Cerium-containing bioactive glass interfaces scavenge ROS and suppress HIF-1alpha-associated inflammatory programs while increasing pro-reparative macrophage infiltration (Cai et al., 2026). ROS-responsive hydrogels release cargo preferentially in oxidatively stressed environments (He et al., 2026; Zheng et al., 2026). EPO-loaded nanoparticles can restore mitochondrial integrity and suppress cGAS-STING signaling during Achilles tendon regeneration (Liu Y. et al., 2026). Together, these studies support metabolic and redox reprogramming as tractable design principles, while the evidence remains predominantly preclinical and varies in proximity to rotator cuff disease.

### 5.6 Mechanosensing: why rehabilitation and material mechanics are immune signals

Macrophages sense matrix mechanics. Cytoskeletal remodeling allows macrophages to detect extracellular-matrix properties and activate tissue-repair gene programs (Meizlish et al., 2024). This principle is highly relevant to the shoulder, where repaired tissue experiences rapidly changing strain, stiffness, and fluid shear during rehabilitation. Mechanical stimulation can itself alter macrophage polarization and IL-4/JAK/STAT signaling in rotator cuff repair (Liu et al., 2022), demonstrating that rehabilitation is not only a mechanical dose to tendon cells—it is also an immune intervention.

Material stiffness, fiber alignment, porosity, surface chemistry, and degradation products similarly act as immune cues. Therefore, the immunological performance of a scaffold cannot be reduced to whether it is “biocompatible.” A high-impact material study should quantify how physical properties alter immune-state trajectories and whether those immune changes causally improve fibrocartilage formation and mechanical function (Wan et al., 2024; Wei et al., 2023; Xu et al., 2023; Sheng et al., 2025).

Human cuff tissue defines disease-associated macrophage states (Qin et al., 2026); rotator cuff models support selected macrophage interventions and mechanosensitive signaling (Liu et al., 2022; He et al., 2025). Efferocytosis mechanisms are supported by non-cuff tendon–bone perturbation (Yao et al., 2026; Zhao et al., 2026), while immunometabolic pathways remain predominantly extrapolated from broader biology or preclinical tendon experiments (Ngai et al., 2023; Cai et al., 2026; Liu Y. et al., 2026). Neither clinical efficacy nor a human cuff causal chain has been demonstrated for these targets (Figure 2).

**FIGURE 2:**
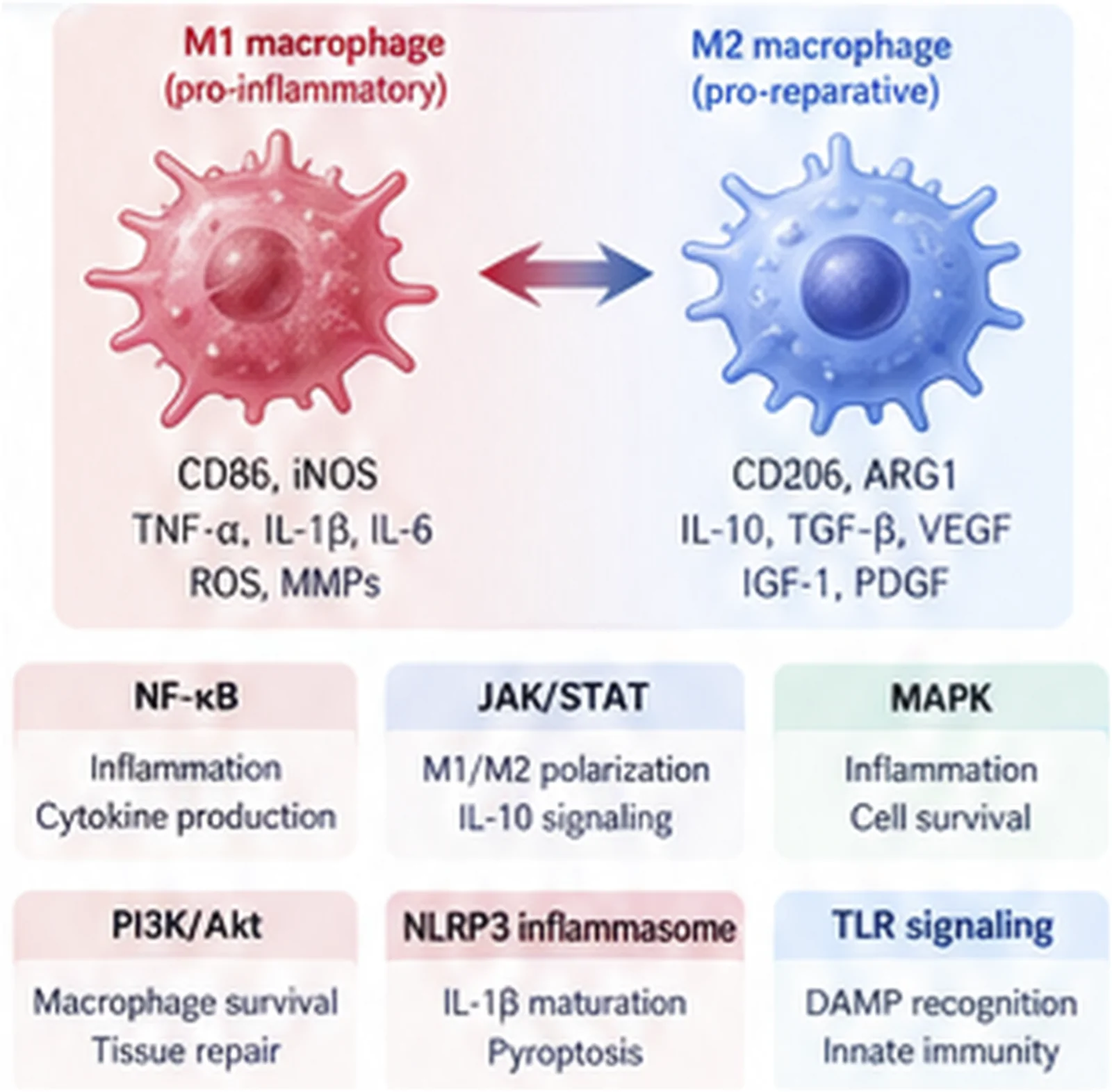
Macrophage dynamics and key signaling pathways in rotator cuff healing. Classical inflammatory and pro-reparative markers are retained as orientation points, but macrophage behavior is presented as a continuum shaped by origin, efferocytosis, metabolism, redox state, matrix mechanics, and spatial niche.

## 6 Immune–stromal cross-talk across the muscle–tendon–bone unit

### 6.1 Tenocytes and tendon stem/progenitor cells

Tenocytes and tendon stem/progenitor cells (TSPCs) are not passive recipients of immune cytokines. They express pattern-recognition receptors, respond to inflammatory mediators, alter extracellular-matrix synthesis, and release signals that feed back onto immune cells. Prolonged inflammatory exposure can drive catabolism, senescence, and loss of tenogenic capacity. Foundational studies established the existence and multipotency of TSPCs and showed that age and disease alter tendon-cell behavior (Bi et al., 2007; Pauly et al., 2010; Randelli et al., 2013; Utsunomiya et al., 2013; Klatte-Schulz et al., 2012; Klatte-Schulz et al., 2013). The newer challenge is to define how immune-state trajectories determine whether these cells regenerate tendon, enter senescence, or adopt maladaptive fibrocartilaginous/osteogenic states.

Aged chronic rotator cuff tears offer a compelling example. Senescent tendon stem cells can promote inflammatory macrophage polarization, and macrophage-conditioned signals can in turn accelerate tendon-cell senescence, forming a self-reinforcing loop. Healthy TSPC-derived exosomes can interrupt this loop, shift macrophages toward a pro-reparative phenotype, and improve tendon-to-bone healing (Zhang X. et al., 2024). This type of reciprocal feedback is more biologically plausible than a one-way “macrophage regulates stem cell” model and should guide future therapeutic design.

### 6.2 Mesenchymal stromal cells, chondrogenesis, and osteogenesis

Mesenchymal stromal cells (MSCs) have long been investigated for rotator cuff augmentation because they can provide paracrine, immunomodulatory, chondrogenic, and osteogenic signals (Voss et al., 2016; Hernigou et al., 2015; Gulotta et al., 2009; Hernigou et al., 2014; Mazzocca et al., 2011; Mazzocca et al., 2010). The current view emphasizes reciprocal communication: MSCs sense inflammatory cytokines and can suppress or redirect immune activation, while macrophage-derived cytokines alter MSC survival, migration, and lineage commitment (Chen et al., 2023). This bidirectional interaction is particularly important at the enthesis, where successful repair requires fibrocartilage and mineral integration rather than indiscriminate osteogenesis.

Macrophage-derived TGF-β1 can promote chondrogenesis under mechanical loading (Wang et al., 2023), whereas persistent inflammatory macrophages can suppress osteogenic and chondrogenic differentiation. Ion-releasing materials exploit this cross-talk: magnesium- and manganese-containing systems can modify macrophage behavior while simultaneously supporting osteochondral lineage programs (Cheng et al., 2022). The strongest designs therefore do not treat immune modulation and tissue differentiation as separate modules; they use a common material cue to couple them.

### 6.3 Fibro-adipogenic progenitors and the immune basis of muscle degeneration

Rotator cuff outcome is strongly influenced by muscle quality. After chronic tendon detachment, muscle atrophy, fibrosis, and intramuscular fat can persist despite technically successful repair. FAPs are a major cellular source of these pathological tissue changes and sit at an immunometabolic-mechanical crossroads (Zhou et al., 2024; Agha et al., 2021). Macrophages can redirect FAP fate through secreted factors and extracellular vesicles: exosomes from macrophages classified as M2 by the original investigators promote beige/brown adipogenic programs and reduce atrophy and fatty degeneration in experimental rotator cuff injury (Liu et al., 2023). Conversely, persistent inflammatory signaling and altered mechanical input favor pathological FAP expansion or adipogenesis. This crosstalk helps explain why enthesis-focused therapies may fail to restore function if the muscle niche remains biologically diseased.

Recent work strengthens the mechanobiological arm of this model across human tissue profiling and rotator cuff animal validation. Human and murine FAP analyses showed that rotator cuff tear suppresses PIEZO1 signaling and increases adipogenic competence; restoring mechanosensitive PIEZO1 activity with low-intensity pulsed ultrasound reduced FAP adipogenesis and improved shoulder function *in vivo* (Lin et al., 2025). Pharmacological studies also show that FAP adipogenesis can be reduced by metformin through mTOR/ULK1-associated autophagy (Zhang et al., 2025). These findings connect mechanical sensing, stromal fate, and muscle degeneration, while therapeutic efficacy remains supported primarily by preclinical perturbation.

### 6.4 Vascular and endothelial cross-talk

Angiogenesis is essential for nutrient delivery and cell recruitment but must be spatially and temporally controlled. Excessive or persistent neovascularization can accompany chronic tendinopathy, whereas inadequate vascular support limits repair. Macrophages regulate endothelial behavior through VEGF, PDGF, TGF-β, and extracellular vesicles. Bioactive-glass-elicited EVs can couple a macrophage marker pattern termed M2 in the original study with angiogenesis (Xu et al., 2023), and multifunctional hydrogels can simultaneously suppress NF-κB and increase VEGF-mediated vascularization (Wang et al., 2025). Human single-cell data also reveal endothelial-to-mesenchymal transition in fibrotic rotator cuff scars, indicating that endothelial cells can become pathological matrix contributors rather than simple vascular conduits (Qin et al., 2026).

### 6.5 Bone, osteoclast-lineage cells, and the immune–skeletal interface

Bone-side healing requires coordinated osteoblast, osteoclast, vascular, and immune activity. Macrophages and osteoclast-lineage cells share myeloid origins and respond to many of the same cytokines. Excessive inflammatory signaling can impair bone formation, whereas IL-10-dependent macrophage immunomodulation can promote osteogenesis in magnesium-based systems (Chen L. et al., 2025; Xiao et al., 2025). This matters clinically because poor bone quality, osteoporosis, and advanced age compromise anchor fixation and bone-side integration. Materials that combine immune regulation with controlled osteogenic support may therefore be especially valuable in high-risk patients (Song et al., 2023; Kong et al., 2026) (Figure 3).

**FIGURE 3:**
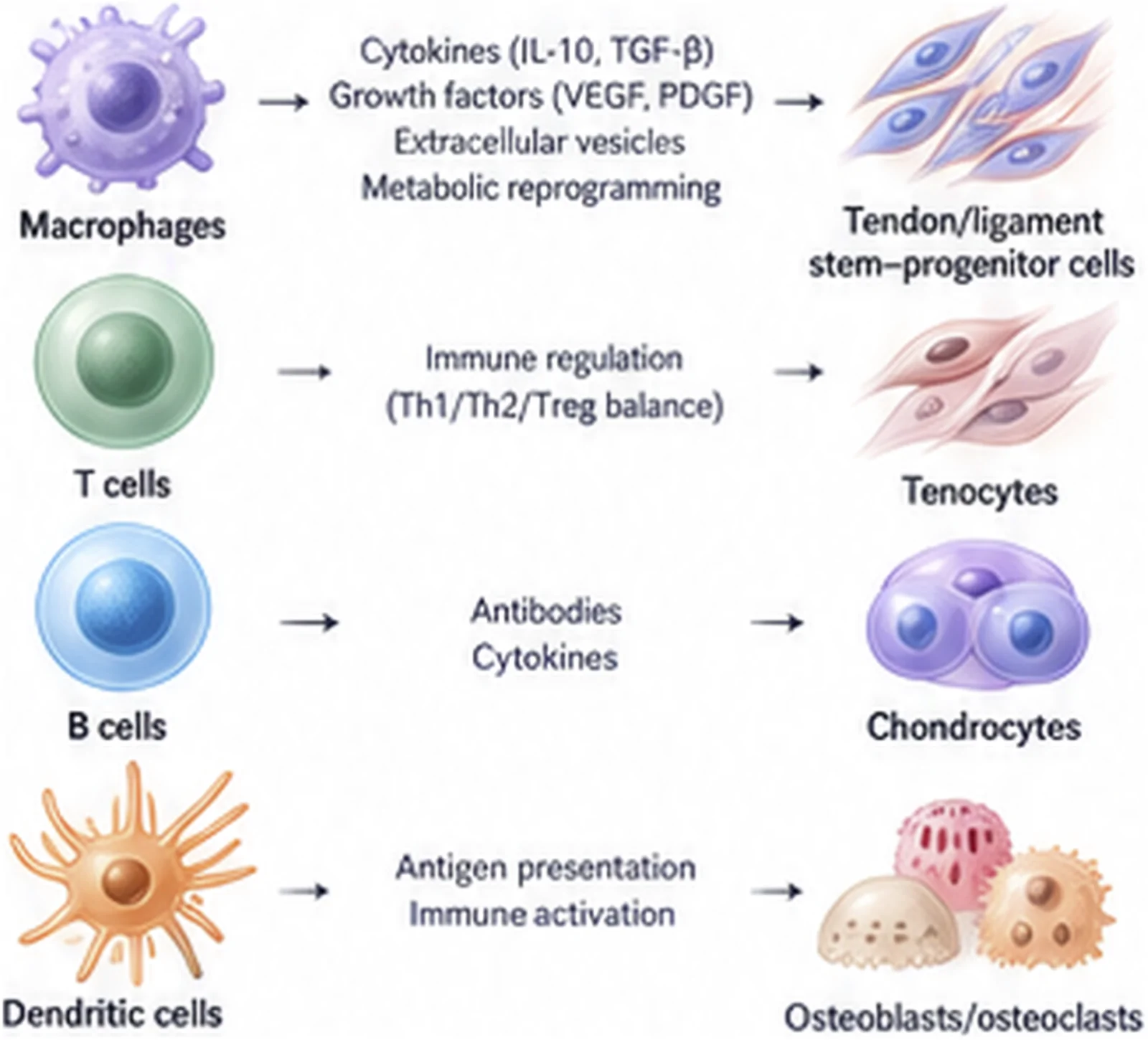
Immune-stromal crosstalk across the rotator cuff muscle-tendon-enthesis-bone unit. Macrophages, lymphocytes, and dendritic cells influence TSPCs/MSCs, tenocytes, FAPs, vascular cells, chondrocytes, and osteoblast/osteoclast-lineage cells, linking immune state to fibrosis, fatty degeneration, fibrocartilage regeneration, angiogenesis, and bone integration.

## 7 Molecular circuits that determine inflammation, resolution, and regeneration

### 7.1 TLR–NF-κB and the IL-1β/TNF-α/IL-6 axis

Pattern-recognition signaling through Toll-like receptors converges on NF-κB and MAPK pathways to induce inflammatory cytokines and chemokines. IL-1β and TNF-α increase matrix metalloproteinase activity, amplify cytokine production, and can suppress tenogenic matrix synthesis when sustained. IL-6 has context-dependent effects on immune recruitment and stromal activation. These pathways are therefore logical targets, but complete blockade is unlikely to be optimal because they also participate in early defense and repair initiation (Crosio and Huang, 2022; Klatte-Schulz et al., 2024; Yang et al., 2025; Chen R. Q. et al., 2025). Responsive systems that suppress NF-κB only when inflammation is excessive may offer a more physiologic solution (Wang et al., 2025; He et al., 2025; He et al., 2026; Zheng et al., 2026).

### 7.2 NLRP3 inflammasome, pyroptosis, and sterile inflammation

NLRP3 can connect DAMP and ROS sensing to IL-1β maturation and pyroptosis in broader immunology, but the cited tendon literature does not yet establish an NLRP3-dependent causal chain in human rotator cuff repair (Klatte-Schulz et al., 2024; Yang et al., 2025; Zhang et al., 2026; Jiang et al., 2024). NLRP3 expression alone is insufficient: rotator cuff experiments should test inflammasome activation, downstream pyroptosis, and pathway-specific perturbation before therapeutic effects are attributed to NLRP3.

### 7.3 IL-4/IL-13–JAK/STAT6 and reparative polarization

IL-4 and IL-13 can activate JAK/STAT6 signaling and promote reparative macrophage programs. Rotator cuff mechanical stimulation improves healing partly through an IL-4/JAK/STAT-associated change in the authors’ M2 marker panel (Liu et al., 2022), and inflammation-responsive micro-hydrogels can deliver IL-4 in a stimulus-dependent fashion (Chen B. et al., 2025). The translational challenge is duration: prolonged type 2 signaling can become fibrotic, particularly in TGF-β-rich environments. The therapeutic objective should therefore be a timed transition, not permanent STAT6 activation.

### 7.4 TGF-β/SMAD: regeneration versus fibrosis

TGF-beta is one of the most context-dependent pathways in tendon healing. It supports chondrogenesis, matrix synthesis, and wound closure, but persistent or spatially inappropriate signaling can activate myofibroblasts, FAP fibrosis, and scar formation (Wang et al., 2023; Schulze-Tanzil et al., 2022; Nakamura et al., 2026; Li et al., 2023). Human rotator cuff single-cell data now place this ambiguity directly in the disease tissue: osteopontin/TGF-beta activity links pro-fibrotic macrophages, senescent tenocytes, and endothelial-to-mesenchymal transition to extracellular-matrix deposition (Qin et al., 2026). Thus, the therapeutic question is not whether TGF-beta should be increased or inhibited globally, but which cell source, isoform, anatomical compartment, and time window should be targeted. Local and temporally gated modulation is biologically more defensible than systemic blockade.

### 7.5 CCL2–CCR2 and monocyte recruitment

CCL2–CCR2 controls monocyte recruitment and is an attractive target because chronic influx of inflammatory monocytes can sustain fibrosis. Yet CCR2 is also expressed by tendon-resident immune cells and is required for aspects of normal healing (Muscat et al., 2022). Pharmacological CCR2 antagonism can improve selected regenerative outcomes when recruitment is excessive (Smolyak et al., 2025). This apparent contradiction exemplifies the need for temporal precision: the same pathway can be required early and detrimental later.

### 7.6 ROS–HIF-1α–Nrf2 and immunometabolic stress

Oxidative stress is a central feature of the hostile repair microenvironment. ROS amplify NF-κB and inflammasome signaling, damage mitochondria, alter progenitor differentiation, and can stabilize HIF-1α. Redox-responsive biomaterials are therefore particularly attractive because the pathological cue itself can trigger treatment. Cerium-doped bioactive glass, metal–phenolic nanoparticles, and ROS-sensitive boronate or thioether chemistries have been used to scavenge ROS, reprogram macrophages, and support fibrocartilage/osteogenic regeneration (Chen et al., 2020; Chen et al., 2021; Huo et al., 2026). Nrf2-focused strategies are comparatively underdeveloped in rotator cuff repair and represent a useful future direction.

### 7.7 cGAS–STING, mitochondrial damage, and innate sensing

Mitochondrial injury can release DNA into the cytosol and activate cGAS–STING signaling, linking metabolic stress to innate inflammation. EPO delivery in tendon regeneration has been reported to restore mitochondrial integrity, promote pro-reparative macrophage polarization, and suppress cGAS–STING signaling (Liu Y. et al., 2026). Similar pathways may be relevant to aged rotator cuff tissue, in which mitochondrial dysfunction and senescence-associated signals are common. Targeting mitochondrial quality control could therefore complement extracellular redox scavenging.

### 7.8 IL-33/Treg signaling and type-2 immune transition

IL-33 is a DAMP-like cytokine with highly context-dependent functions. Regenerative neonatal tendon healing is associated with Treg-dependent regulation of IL-33 signaling, and adult IL-33 deletion can improve functional healing in experimental models (Arvind et al., 2025; Voss et al., 2021). These findings suggest that successful repair requires not simply more or less IL-33 but correct coordination of type 1 and type 2 immune phases. Biomaterials capable of recruiting or programming Tregs could provide a route to this higher-order immune control (Shen et al., 2021; Nayer et al., 2024; Dwyer et al., 2022; Ahn et al., 2025; Nie et al., 2024).

### 7.9 Specialized pro-resolving mediators

Resolution is an active biochemical process mediated in part by lipid-derived specialized pro-resolving mediators (SPM), not merely the passive disappearance of inflammatory cytokines. Human subacromial bursa contains SPM and therefore may participate in endogenous resolution signaling (Klatte-Schulz et al., 2024). This observation is strategically important because most rotator cuff biomaterials are still designed around anti-inflammatory cytokine suppression or induction of a limited CD206/ARG1-associated marker set. A more physiological approach would test whether treatment restores pro-resolving lipid programs, apoptotic-cell clearance, and immune-cell exit while preserving early host-defense functions. Direct manipulation of SPM pathways in rotator cuff repair remains underdeveloped and represents a tractable gap between human tissue observations and therapeutic design.

Human cuff samples implicate TGF-β-linked scar states and show bursal pro-resolving mediators (Qin et al., 2026; Klatte-Schulz et al., 2024). Rotator cuff model interventions support roles for IL-4-associated signaling and macrophage-derived TGF-β1 (Wang et al., 2023; Liu et al., 2022); evidence for NLRP3-dependent repair, cGAS–STING, IL-33/Tregs, and efferocytosis is mainly from other tendon models or broader biology (Arvind et al., 2025; Liu Y. et al., 2026; Yao et al., 2026; Zhao et al., 2026). Those mechanisms remain rotator cuff hypotheses until directly tested.

## 8 Disease modifiers that reshape the immune niche

### 8.1 Aging, senescence, and inflammaging

Rotator cuff disease is strongly age associated, and aging alters both resident tendon cells and immune competence. Older tenocyte-like cells show reduced proliferative and anabolic responses (Klatte-Schulz et al., 2012; Klatte-Schulz et al., 2013), while senescent TSPCs can produce a secretory phenotype that reinforces inflammatory macrophage activity. The senescent-TSPC/macrophage positive feedback observed in aged chronic tears provides a mechanistic explanation for why regenerative therapies that succeed in young animals may fail in elderly patients (Zhang X. et al., 2024). Senomorphic extracellular-vesicle/hydrogel strategies represent an emerging attempt to modify this disease state rather than merely augment the repair mechanically (Kong et al., 2026).

### 8.2 Obesity, adipokines, and metabolic inflammation

Obesity and fatty degeneration correlate with inferior rotator cuff outcomes, but the link is not purely mechanical. Leptin can directly enhance an inflammatory macrophage profile designated M1 in the original study through TNF-related signaling and worsen tendon-to-bone healing (Li Y. et al., 2025). This observation suggests that systemic metabolic phenotype can alter local immune trajectories even when the surgical repair is technically identical. Future patient stratification should therefore consider metabolic biomarkers, body composition, and muscle fat as immune-relevant variables rather than generic comorbidities.

### 8.3 Diabetes and impaired resolution

Diabetes is associated with altered collagen crosslinking, vascular dysfunction, oxidative stress, and impaired immune resolution. Although the rotator cuff-specific immunology of diabetes remains incompletely mapped, diabetic tendon displays altered cellular and matrix characteristics and poorer healing. Therapies that rely on macrophage reprogramming may behave differently in hyperglycemic tissue because baseline metabolism, ROS production, and cytokine responsiveness are altered. Diabetic and metabolically stressed models should therefore be included before claiming broad clinical applicability.

### 8.4 Osteoporosis and poor bone-side integration

Osteoporosis changes both mechanics and immune biology at the bone side of the enthesis. Osteoporotic marrow can contain altered stromal and myeloid populations, and poor bone stock magnifies the consequences of incomplete fibrocartilage formation. Aligned macroporous hydrogels with small extracellular vesicles and senomorphic vesicle–hydrogel systems have been developed specifically for osteoporotic tendon-to-bone healing (Song et al., 2023; Kong et al., 2026). These approaches exemplify disease-modifying biomaterials: they aim to correct a hostile host phenotype, not simply add a generic regenerative cue.

### 8.5 Chronicity, denervation, and fatty degeneration

Chronic tears combine mechanical unloading, denervation, muscle retraction, FAP expansion, fibrosis, and altered macrophage signaling. Acute rodent repair models reproduce only a fraction of this biology. Delayed repair and muscle-denervation models reveal the persistence of FAP and immune abnormalities and provide a more stringent test for therapies (Liu et al., 2023; Zhou et al., 2024; Agha et al., 2021; Mazzocca et al., 2010; Oh et al., 2014; Gumucio et al., 2016; Chen et al., 2015; Uysal et al., 2012; Pfaff et al., 2025; Gui and Meyer, 2024; Bowen et al., 2025; Merriman et al., 2025). A treatment intended for massive chronic tears should therefore demonstrate efficacy in chronic disease, not merely in an acute detachment immediately repaired in young animals.

### 8.6 Sex, endocrine state, and immune-metabolic context

Sex and endocrine state are likely to modify the immune-regenerative niche but are rarely built into rotator cuff biomaterial studies. FAPs isolated from female rotator cuff tissue can show distinct autophagic and adipogenic behavior, and metformin-sensitive mTOR/ULK1 signaling has been implicated in sex-associated differences in fatty degeneration (Zhou et al., 2024). Menopause, adipokine signaling, osteoporosis, and age-related immune remodeling frequently coexist in the same clinical population, suggesting that ‘female sex,’ ‘obesity,’ and ‘osteoporosis’ should not be modeled as independent variables. Future work should therefore stratify sex, age, bone quality, and metabolic state and should test whether the same immunomodulatory therapy has different effects across these biological contexts. This is particularly important for therapies that act through estrogen-sensitive immune pathways, adipokines, macrophage metabolism, or senescence.

## 9 Immunomodulatory regenerative therapies

### 9.1 Cytokines, chemokines, and small molecules

Direct immune modulation can target cytokines, chemokines, intracellular pathways, and metabolites, but exposure and tissue context can reverse the intended effect. Early blockade of CCR2 or inflammatory mediators may diminish debris clearance; sustained IL-4/type-2 or TGF-β-rich signaling may sustain fibroblast activation, FAP-mediated fibrosis, or scar rather than enthesis regeneration (Wang et al., 2023; Muscat et al., 2022; Smolyak et al., 2025; Liu et al., 2022; Chen B. et al., 2025). A change in M2 markers is therefore insufficient evidence of benefit. Dose, onset after repair, duration, and tendon-, muscle-, or bone-side location should be tested against early host response, fibrosis, fibrocartilage organization, and mechanics.

### 9.2 Platelet-rich plasma and marrow-derived orthobiologics

PRP is already used during arthroscopic repair, and randomized human studies measure imaging-confirmed retears and patient outcomes. Yet results depend on preparation, leukocyte content, tear size, dosing, and follow-up: in one double-blind trial, intraoperative leukocyte-poor PRP reduced 6-month MRI retear without improving patient-reported function (Rossi et al., 2024); another three-arm trial found no structural or functional advantage of either leukocyte-rich or leukocyte-poor PRP at 12 months (Yao et al., 2024). These clinical findings do not establish that macrophage regulation mediated an effect. PRP products should report cellular composition and activation, while proposed immunomodulation needs tissue-specific measurements. Bone marrow aspirate concentrate and other orthobiologics should not inherit PRP’s clinical evidence by association.

### 9.3 Cell-based therapies

Cell therapies from marrow, adipose, tendon, and bursa have been investigated in shoulder or tendon repair (Utsunomiya et al., 2013; Voss et al., 2016; Hernigou et al., 2015; Gulotta et al., 2009; Hernigou et al., 2014; Mazzocca et al., 2011; Mazzocca et al., 2010; Meyer et al., 2015; Eliasberg et al., 2017; Steinert et al., 2015), but cell source and processing vary. An *ex vivo* anti-inflammatory response does not show durable engraftment or prove healing in a chronic tear. Cell expansion, donor age, batch variation, contamination control, viability after storage, and arthroscopic handling must be addressed; persistent paracrine TGF-β/type-2 cues may also favor fibrosis. Human surgical use of some cellular adjuncts does not validate a specific immune mechanism or support treating all products as one class.

### 9.4 Extracellular vesicles and engineered exosomes

EVs offer cell-free delivery of immunoregulatory cargo, with animal rotator cuff studies reporting improvements in histology, interface mechanics, or muscle quality (Zhang X. et al., 2024; Han et al., 2024; Tong et al., 2023; Song et al., 2024; He et al., 2024; Qin et al., 2024; Tang et al., 2025; Aratikatla et al., 2025; Zou et al., 2023; Kim et al., 2022; Zhang J. et al., 2024). The aged chronic rat model in (Zhang X. et al., 2024) is particularly relevant, but its favorable early postoperative macrophage shift cannot be generalized to every disease phase, donor, or recipient. EV cargo and cell source vary with donor age and conditioning; an immunosuppressive product could hinder early clearance, whereas an osteogenic or pro-fibrotic cargo could be harmful in the wrong compartment. Human macrophage–tenocyte assays (von et al., 2025) show source-dependent biology but do not establish patient efficacy.

Before clinical testing, engineered EVs require defined identity and active cargo, potency and dose assays, purity and sterility controls, stability after storage, off-target biodistribution, and product-specific immunogenicity assessment (Song et al., 2024; He et al., 2024; Aratikatla et al., 2025; Zou et al., 2023). A hydrogel carrier can prolong exposure and change recipient-cell access, so carrier-alone and EV-alone controls, degradation studies, and delayed/chronic disease models are essential. No cited engineered EV system has demonstrated clinical rotator cuff repair efficacy.

### 9.5 Immunomodulatory biomaterials

Biomaterials couple spatial organization to immune control through chemistry, stiffness, porosity, fiber architecture, degradation, ion release, and biologic cargo (Chen Y. et al., 2024; Zhu et al., 2025). Acellular collagen patch augmentation already has human surgical studies with retear outcomes (Hurley et al., 2024; Warren et al., 2024; de Andrade et al., 2022), but those outcomes do not establish the immunomodulatory mechanism claimed for newer responsive scaffolds. Material-linked macrophage changes can be context dependent: prolonged type-2 cues may sustain fibrosis, excessive osteogenic or ion release may cause ectopic mineralization or local toxicity, and changing stiffness can alter both immune behavior and repair mechanics. Each construct should isolate its material and immune contributions (Chen Z. et al., 2025).

ROS-responsive hydrogels and antioxidant or ion-releasing suture coatings have produced encouraging histological and, in selected rat rotator cuff studies, biomechanical outcomes (Cai et al., 2026; Wang T. et al., 2024; Chen B. et al., 2025; Huo et al., 2026). Their activation threshold may perform differently in chronic, aged, diabetic, or low-ROS tissue; excessive ROS scavenging could also blunt early defense signaling. For injectable or suture-mounted products, bench tests must establish handling through a portal, fixation under irrigation, retention during early loading, release after sterilization and storage, and long-term degradation safety. Delivery plausibility in rodents is not proof of arthroscopic feasibility in patients.

Multi-component designs should include carrier-only, cargo-only, and ablation controls and show that immune-state change mediates improved fibrocartilage and fatigue resistance. Senomorphic EV/hydrogel studies in osteoporotic rotator cuff rats (Kong et al., 2026) and immunometabolic/mitochondrial strategies in tendon models (Cai et al., 2026; Liu Y. et al., 2026) are biologically informative, but suppressing senescence-associated signaling too early may perturb normal wound responses, and metabolic interventions may differ with diabetes or host age. These adverse effects are plausible risks requiring direct measurement, not harms already established for the cited products. Human tissue validation, systemic exposure, and manufacturing reproducibility remain open.

### 9.6 Physical stimulation and rehabilitation as immunomodulation

Mechanical loading is also an immune signal: it alters macrophage state, TGF-beta signaling, vascular behavior, and matrix organization. Eccentric stimulation can improve rotator cuff healing through macrophage regulation (Peng et al., 2026), whereas excessive early loading may perpetuate damage. Rehabilitation should therefore be studied as part of the immunomodulatory regimen, with loading dose and timing linked to immune and mechanical outcomes.

PRP and some acellular patches have human rotator cuff outcome studies, but their specific immune mechanisms are not clinically established (Hurley et al., 2024; Rossi et al., 2024; Yao et al., 2024). The EV, senomorphic, ROS-responsive, ion-releasing, and immunometabolic interventions are mainly preclinical (He et al., 2025; Cai et al., 2026; Liu Y. et al., 2026; Kong et al., 2026; Chen B. et al., 2025; Zhang X. et al., 2024; Huo et al., 2026). Human bursal signaling (Klatte-Schulz et al., 2024) and human tendon primary-cell EV experiments (von et al., 2025) supply biological context, not treatment efficacy (Figure 4). Table 6 applies translation criteria consistently across these approaches.

**FIGURE 4:**
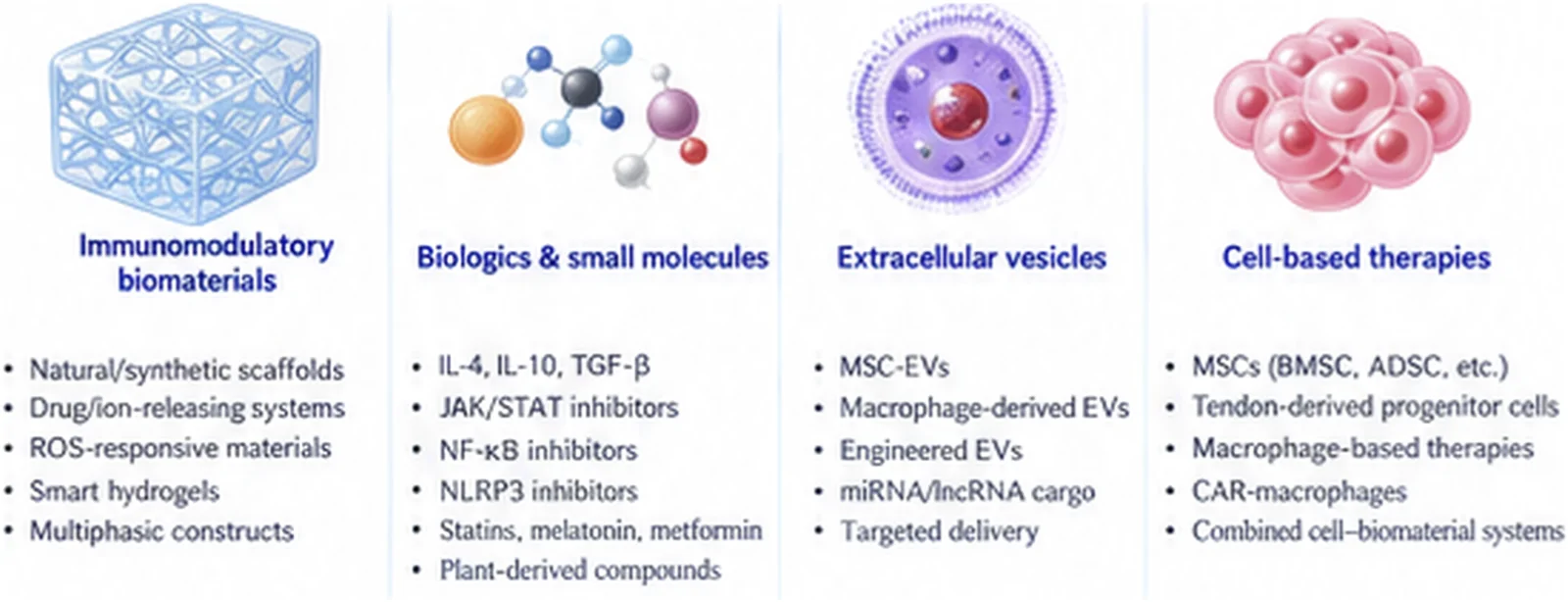
Immunomodulatory regenerative strategies. The field is progressing from constitutive anti-inflammatory delivery toward pro-resolution, responsive, cell-targeted, and spatiotemporally staged systems that integrate biomaterials, extracellular vesicles, biologics, cell therapy, and mechanical rehabilitation.

**TABLE 6:** Historical evidence base that underpins contemporary immunomodulatory rotator cuff strategies.

Evidence stream	Contribution to current immune-regenerative framework	Refs
Stem/progenitor-cell augmentation	Cellular/paracrine augmentation with variable translation	(Bi et al., 2007; Randelli et al., 2013; Utsunomiya et al., 2013; Voss et al., 2016; Gulotta et al., 2009; Hernigou et al., 2014; Mazzocca et al., 2010; Oh et al., 2014; Meyer et al., 2015; Eliasberg et al., 2017; Steinert et al., 2015)
ECM, collagen, fibrin and biologic scaffolds	Matrix delivery and host-cell infiltration; exposes processing and immune-response limitations	(Derwin et al., 2010; Ricchetti et al., 2012; Chainani and Little, 2016; Longo et al., 2013; Voss et al., 2016; Kim et al., 2022; Chen et al., 2024b; Zhu et al., 2025; Li et al., 2025b; Codispoti et al., 2024; Kaizawa et al., 2019; Qian et al., 2019)
Growth-factor delivery	Dose, release kinetics, and ectopic-response risk	(Wang et al., 2023; Chen et al., 2020; Rodeo et al., 2007; Seeherman et al., 2008; Tokunaga et al., 2015; Huang et al., 2021; Prabhath et al., 2018)
Synthetic and hybrid reinforcement	Mechanical reinforcement; highlights foreign-body and biological limitations	(Chen et al., 2024b; Zhu et al., 2025; Huang et al., 2021; Zou et al., 2025; Wu et al., 2024; Ping et al., 2026; Zhu et al., 2026; Fang et al., 2026; Dong et al., 2024; Zhang et al., 2024b)
Modern gradient/bilayer/patch strategies	Shift toward interface-specific design, clinically deployable augmentation, and evidence synthesis	(Chen et al., 2024b; Zhu et al., 2025; Li et al., 2025b; Codispoti et al., 2024; Zou et al., 2025; Hurley et al., 2024; Feng et al., 2025; Warren et al., 2024; Cobb et al., 2022; de Andrade et al., 2022)
Disease-modifying and EV-enabled systems	Osteoporosis, senescence, redox stress, and cell-free signaling are emerging as high-risk-specific targets	(Song et al., 2023; Kong et al., 2026; Zhang et al., 2024c; Zhou et al., 2024; Lin et al., 2025; Pfaff et al., 2025; Han et al., 2024; He et al., 2024; Kim et al., 2022; Zhang et al., 2024a)

The table also provides continuity between classic scaffold work and current immune-regenerative engineering.

## 10 Design principles for next-generation immune-regenerative therapies

### 10.1 Replace “anti-inflammatory” with “pro-resolution”

Pro-resolution therapies should be tested with serial functional readouts, including clearance, inflammatory persistence, and tissue reconstruction, alongside structural and mechanical healing outcomes.

### 10.2 Target state transitions, not static phenotypes

The therapeutic target should be a trajectory: inflammatory competence → resolving macrophage programs → progenitor instruction → fibrocartilage formation → remodeling homeostasis. This can be achieved through time-dependent dosing, degradable compartments, enzyme-triggered release, ROS-responsive chemistry, or rehabilitation-linked mechanical stimulation (Liu et al., 2022; Wang et al., 2025; Cheng et al., 2025; Chen B. et al., 2025). A static depot that continuously releases IL-4 or another reparative cytokine may be less physiologic than a system whose activity decays after the transition has occurred.

### 10.3 Treat the rotator cuff as a muscle–tendon–bone organ

For chronic large tears, success depends on more than tendon–bone integration. Muscle fatty degeneration and fibrosis can limit functional recovery even when imaging shows an intact repair. Immune–FAP regulation, vascular support, and anti-senescence strategies may therefore need to accompany enthesis regeneration (Kong et al., 2026; Liu et al., 2023; Zhou et al., 2024; Agha et al., 2021; Pfaff et al., 2025). This is particularly important for clinical trials, in which structural integrity and patient-reported function can diverge.

### 10.4 Build spatial specificity into therapy

The tendon side, fibrocartilaginous interface, bone side, muscle, and bursa have different immune and regenerative needs. Spatial specificity can be created through multilayer scaffolds, regionally immobilized ligands, local suture coatings, microneedles, or compartment-selective injection(Gao et al., 2023). The clinical design objective should be the minimum spatial complexity needed to solve the dominant biological problem, not maximal architectural novelty (Wan et al., 2026).

### 10.5 Make complexity mechanistically auditable

Each added component should have a defined role and measurable contribution. Factorial or component-ablation experiments should link material feature, immune-state change, stromal response, tissue regeneration, and mechanics. If a simpler construct produces the same biological effect, added complexity is difficult to justify translationally.

### 10.6 Match each claim to its disease context, causal support, and translational stage

A claim should specify all three dimensions where relevant. Human tissue atlases and bursal profiling support disease relevance but generally remain observational (Qin et al., 2026; Klatte-Schulz et al., 2024; Ju et al., 2024); controlled rotator cuff interventions can strengthen causal interpretation in a model without showing clinical benefit (Wang et al., 2023; Liu et al., 2022; He et al., 2025); non-rotator-cuff tendon experiments may establish a pathway yet require explicit extrapolation to shoulder repair (Arvind et al., 2025; Liu Y. et al., 2026; Yao et al., 2026; Zhao et al., 2026). Table 4 records these dimensions separately to prevent proximity to human disease from being mistaken for causal strength or clinical readiness.

## 11 Emerging technologies that can redefine the field

### 11.1 Single-cell and spatial multi-omics

Single-cell RNA sequencing has already exposed macrophage and stromal heterogeneity in rotator cuff scars and in tendon healing more broadly (Cheng et al., 2025). Spatial transcriptomics and proteomics are the logical next step because they preserve the relationship between immune cells and tendon, fibrocartilage, bone, vessels, and muscle. A future “immune atlas” of rotator cuff healing should map cell state, ligand–receptor interactions, mechanical environment, and histological zone across time.

### 11.2 Human tissue and *ex vivo* models

A recurring translational weakness is overreliance on young rodents. Human torn tendon, bursa, muscle, marrow, and repair scar should be incorporated earlier in the development pipeline. *Ex vivo* co-culture systems combining human macrophages, tenocytes, TSPCs, FAPs, and endothelial cells can test whether a therapy reproduces the expected immune–stromal mechanism in diseased human tissue. Primary human macrophage/tenocyte studies already demonstrate that EV effects depend on cell of origin (von et al., 2025).

### 11.3 Organoids, microphysiological systems, and mechanical bioreactors

No existing *in vitro* system reproduces the mechanical and spatial complexity of the enthesis. Microphysiological platforms that combine aligned tendon-like matrix, fibrocartilage, mineralized substrate, perfusion, macrophages, and cyclic loading could provide a more predictive intermediate between monolayer culture and animal studies. Such systems would also enable controlled testing of sequence-dependent immunomodulation and rehabilitation protocols.

## 12 Discussion: translational roadmap for immune-regenerative therapy

### 12.1 Use models that reproduce the intended patient

The highest-risk patients are older, have chronic tears, muscle degeneration, poor tendon quality, metabolic comorbidity, or osteoporosis. Acute repair in young rats is therefore an optimistic screening model rather than evidence of clinical readiness. A rational pipeline should progress from mechanistic small-animal studies to delayed/chronic or disease-modified models and then to large-animal shoulders with clinically relevant loading (Derwin et al., 2010; Chen B. et al., 2025; Dong et al., 2024).

### 12.2 Couple immune endpoints to structural and mechanical outcomes

A therapy should not be declared successful because it increases CD206 staining. Immune changes must co-localize with improved fibrocartilage organization, collagen alignment, mineral gradient, vascular pattern, and ultimately mechanics. Ultimate load to failure is useful but insufficient; cyclic fatigue, stiffness, gapping, and time-dependent degradation are more relevant to rehabilitation. Spatial histology and local mechanical mapping should be integrated whenever possible.

### 12.3 Design around arthroscopy, sterilization, storage, and manufacturing

Arthroscopic use is already documented for PRP and selected acellular patches (Hurley et al., 2024; Rossi et al., 2024; Yao et al., 2024). For newer hydrogels, coated sutures, cells, or EV depots, compatibility remains to be demonstrated through portal delivery, fixation under fluid irrigation, delivery time, and stability under postoperative loading. Sterilization, shelf life, release after storage, batch reproducibility, and potency assays should be reported before an intervention is called surgically deployable (Cai et al., 2026; Du et al., 2024; Sheng et al., 2025; Zou et al., 2023).

### 12.4 Safety: immune modulation has non-linear risks

Immune modulation carries non-linear risks. Early suppression may compromise debris clearance, while sustained IL-4 or TGF-β-associated programs can accompany fibrosis; excess osteogenic cues can encourage heterotopic mineralization. EV cargo can vary with donor, age, or culture, and nanoparticles or degradable scaffolds may produce off-target or foreign-body effects. These are mechanism-based concerns that require direct testing in each product and dosing window; they should not be described as adverse events already demonstrated clinically for the cited experimental products. Relevant assessment includes local and systemic responses, ectopic bone, tendon quality, cyclic mechanics, biodistribution, and longer-term degradation.

### 12.5 Clinical trial design and patient selection

Early trials should enroll patients with sufficient biological risk to justify augmentation, such as large or massive tears, revision repair, poor tendon quality, advanced age, marked fatty degeneration, osteoporosis, or metabolic comorbidity. Comparators should reflect optimized contemporary repair and rehabilitation, and outcomes should include structural healing/retear, pain, strength, patient-reported outcomes, safety, and cost. Biomarker-based enrichment should be prespecified when the intervention targets a defined immune failure mechanism.

### 12.6 Clinically ubiquitous immunomodulation: corticosteroids, NSAIDs, and bursectomy

Corticosteroid injections are among the most clinically relevant immune interventions in rotator cuff disease because they suppress inflammatory signaling while also acting directly on tendon cells and extracellular matrix. Basic-science evidence indicates that corticosteroid exposure can reduce tenocyte proliferation and viability, alter collagen and matrix composition, increase apoptosis, and transiently weaken tendon or repair biomechanics in a dose- and timing-dependent manner (Puzzitiello et al., 2020a). Clinical evidence is more nuanced: preoperative injections have been associated in some observational syntheses with higher revision or infection risk when administered close to repair or repeatedly (Puzzitiello et al., 2020b), whereas postoperative injections given after the earliest healing phase may provide short-term pain and functional benefit without a consistent increase in retear rates (Puzzitiello et al., 2022). Thus, corticosteroids illustrate the central principle of this Review: suppressing inflammation is not equivalent to restoring resolution. Their biological effect is strongly dependent on dose, repetition, compartment, and timing relative to injury and repair.

NSAIDs provide a second clinically ubiquitous test of the same principle. Cyclooxygenase inhibition can reduce prostaglandin-dependent inflammatory signaling and postoperative pain, but animal rotator cuff models have shown impaired tendon-to-bone collagen organization and mechanical strength with both non-selective and COX-2-selective inhibition (Cohen et al., 2006). By contrast, contemporary clinical meta-analyses of arthroscopic rotator cuff repair have not demonstrated a clear increase in retear risk with short postoperative NSAID courses, particularly for non-selective agents, although certainty remains limited and selective COX-2 inhibition may not be biologically equivalent to non-selective treatment (Seah et al., 2023). These apparently divergent data argue against treating “anti-inflammatory therapy” as a single category. Future trials should report drug class, dose, treatment window, and structural healing endpoints, and should test whether early versus delayed cyclooxygenase blockade differentially alters the transition from inflammatory recruitment to resolution and matrix remodeling.

The same reasoning has a surgical analogue in management of the subacromial bursa. Bursectomy may reduce inflamed, pain-generating tissue and improve visualization, but the bursa is also a biologically active compartment containing progenitor and stromal populations with potential regenerative functions (Levy et al., 2022). In a rat rotator cuff tear model, preservation of the subacromial bursa was associated with an early inflammatory program followed by later increases in IL-10, Arg-1, collagen III, neovascularization, and tendon-related repair features (Miura et al., 2024). Together with human evidence for bursal resolution mediators and disease-associated immune heterogeneity discussed above, these findings raise the possibility that indiscriminate bursectomy removes both pathological and potentially pro-reparative signals. Current evidence is insufficient to recommend a specific extent of bursal preservation; rather, prospective human studies should determine whether selective versus extensive bursectomy alters local immune-state trajectories, pain, tendon quality, and structural healing.

### 12.7 Biomarkers, computational integration, and precision immunomodulation

A single circulating cytokine is unlikely to capture the compartmentalized biology of rotator cuff failure. More informative stratification may combine clinical variables (age, tear size, chronicity, fatty degeneration, bone density, and metabolic status) with perioperative tendon, bursal, or marrow features such as macrophage-state programs, senescence burden, osteopontin/TGF-beta activity, and resolution lipid profiles (Qin et al., 2026; Klatte-Schulz et al., 2024; Ju et al., 2024). Computational models can integrate these multidimensional data with material-design parameters, but their value depends on interpretable features, external validation, and prospective testing. A 2026 study illustrates the design potential: a TabPFN-based workflow screened material combinations and selected a staged hydrogel delivering macrophage-targeting small extracellular vesicles (sEV) before kartogenin-mediated chondrogenesis, improving tendon-to-bone repair in rats (Liu W. et al., 2026). Clinically useful biomarkers should ultimately identify a reversible failure mechanism and predict benefit from a matched intervention, rather than serve only as descriptive correlates (Figure 5). The recommended minimum reporting standards for the shoulder cuff immune regeneration study are shown in Table 7.

**FIGURE 5:**
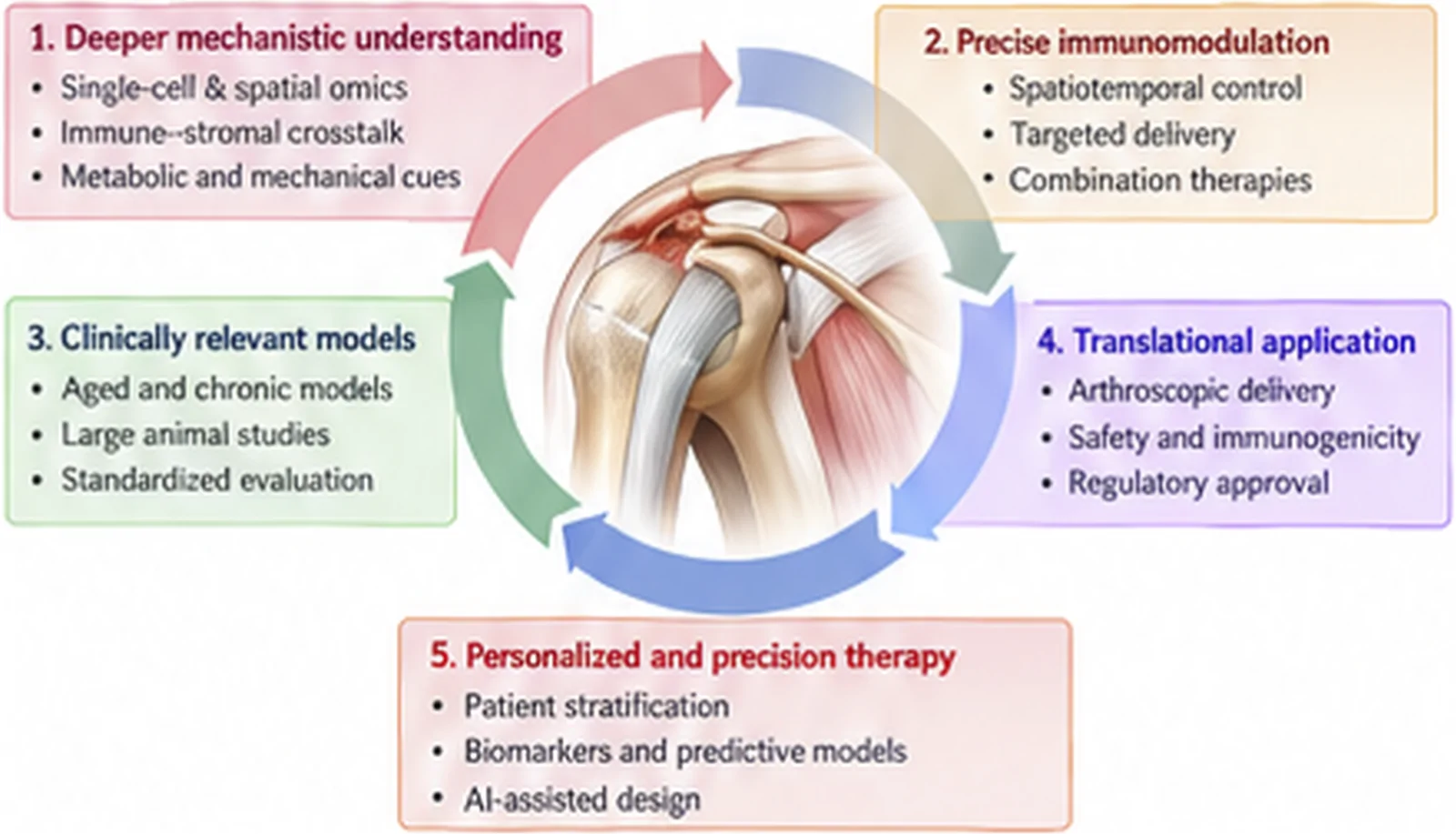
Challenges and future perspectives for precision immune-regenerative rotator cuff therapy. Key priorities are human-defined immune states, causal perturbation, chronic/aged models, surgical deployability, manufacturing discipline, biomarker-guided patient selection, and integration of immune, structural, and mechanical endpoints.

**TABLE 7:** Proposed minimum reporting standard for immune-regenerative rotator cuff studies.

Domain	Minimum reporting recommendation
Immune mechanism	Cell origin/state; time course; ≥2 state markers; functional assay; causal perturbation when feasible
Material immune interaction	Composition, stiffness/topography, degradation, ion/cargo release, sterilization effect
Spatial biology	Tendon, fibrocartilage, bone, muscle and bursa assessed separately when relevant
Regeneration	Collagen alignment, fibrocartilage area, mineralization, vascularity, muscle fibrosis/fat
Mechanics	Ultimate load, stiffness, cyclic fatigue/gapping, failure location
Model fidelity	Age, chronicity, tear size, denervation/fatty degeneration, osteoporosis/metabolic disease
Translation	Arthroscopic deployment, handling, storage, batch reproducibility, safety, regulatory complexity
Clinical relevance	Risk-enriched population, imaging healing, retear, PROMs, strength, adverse events, cost

## 13 Conclusion

Rotator cuff healing is governed by coordinated immune, stromal, vascular, muscle, and bone responses. Human single-cell and bursal studies now identify disease-relevant pro-fibrotic, senescent, endothelial, and macrophage programs, while mechanistic studies nominate efferocytosis, immunometabolism, mechano-immunity, senescence, and immune-stromal crosstalk as tractable control points. The central research task is to move from association to causal, spatially resolved mechanisms in models that reproduce chronic and age-related disease.

Translation should prioritize interventions matched to defined failure mechanisms and patients, using the minimum complexity required for arthroscopic delivery and reproducible manufacturing. Success must connect immune-state change to graded enthesis reconstruction, cyclic mechanical competence, and clinically interpretable function rather than rely on isolated immune markers or histology. This evidence-linked framework provides a practical path from immune biology to durable rotator cuff repair.
